# Dynamic genomic changes in methotrexate-resistant human cancer cell lines beyond *DHFR* amplification suggest potential new targets for preventing drug resistance

**DOI:** 10.1038/s41416-024-02664-0

**Published:** 2024-04-09

**Authors:** Xiang-Ning Meng, Jin-Fa Ma, Yang-He Liu, Si-Qing Li, Xu Wang, Jing Zhu, Meng-Di Cai, Hui-Shu Zhang, Tiantian Song, Shukai Xing, Li-Qing Hou, Huan Guo, Xiao-Bo Cui, Jiang Han, Peng Liu, Guo-Hua Ji, Wen-Jing Sun, Jing-Cui Yu, Song-Bin Fu

**Affiliations:** 1 Key laboratory of preservation of human genetic resources and disease control in China (Harbin Medical University), Ministry of Education, Harbin, 150081 China; 2 Laboratory of Medical Genetics, Harbin Medical University, Harbin, 150081 China; 3 Scientific Research Centre, The Second Affiliated Hospital of Harbin Medical University, Harbin, 150081 China

**Keywords:** Cancer epigenetics, Cancer genomics

## Abstract

**Background:**

Although *DHFR* gene amplification has long been known as a major mechanism for methotrexate (MTX) resistance in cancer, the early changes and detailed development of the resistance are not yet fully understood.

**Methods:**

We performed genomic, transcriptional and proteomic analyses of human colon cancer cells with sequentially increasing levels of MTX-resistance.

**Results:**

The genomic amplification evolved in three phases (pre-amplification, homogenously staining region (HSR) and extrachromosomal DNA (ecDNA)). We confirm that genomic amplification and increased expression of *DHFR*, with formation of HSRs and especially ecDNAs, is the major driver of resistance. However, *DHFR* did not play a detectable role in the early phase. In the late phase (ecDNA), increase in FAM151B protein level may also have an important role by decreasing sensitivity to MTX. In addition, although MSH3 and ZFYVE16 may be subject to different posttranscriptional regulations and therefore protein expressions are decreased in ecDNA stages compared to HSR stages, they still play important roles in MTX resistance.

**Conclusion:**

The study provides a detailed evolutionary trajectory of MTX-resistance and identifies new targets, especially ecDNAs, which could help to prevent drug resistance. It also presents a proof-of-principal approach which could be applied to other cancer drug resistance studies.

## Introduction

Gene amplification, one of the most important forms of somatic genomic instability, has been demonstrated to be a common adaptive response to a number of different selective pressures, such as treatment with chemotherapy drugs [1–4]. Gene amplification can occur either on paired “double minute” forms of extrachromosomal DNAs (ecDNAs) [5] or on aberrant intrachromosomal regions called homogeneously staining regions (HSRs) [6]. Kohl et al. demonstrated that *MYCN* could be mapped to HSRs and ecDNAs in the IMR-32 neuroblastoma cell line [7]. Similar observations were found by Alitalo et al. in neuroendocrine cells derived from a colorectal carcinoma [8]. Previous studies confirmed that ecDNAs represent an “unstable” form of gene amplification, whereas HSRs represent a “stable” one [9, 10]. EcDNA amplification may enable oncogenes or drug resistance genes to rapidly reach high copy numbers because of the unequal segregation to daughter cells [5] and elevate transcriptional level by the specific 3D topologic structure more highly than HSR amplification. Thus, ecDNA-based amplification enables tumors to rapidly acquire and maintain intratumoral genetic heterogeneity, suggesting a central role of ecDNAs in the acceleration of tumor evolution [5].

MTX is commonly used in the treatment of many types of cancers, although the wide development of resistance in different cancers has greatly limited its effectiveness. Defective transportation [11] or decreased retention of the drug [12], changes in translational regulation of *DHFR* [13, 14], increased *DHFR* activity due to gene amplification, and mutant forms of *DHFR* with reduced affinity for MTX have all been found to contribute to drug resistance [15]. Among these, *DHFR* amplification plays a dominant role in acquired MTX resistance, giving rise to HSRs and ecDNAs [15, 16]. Drug resistance presents a real challenge as cancer progression is an evolutionary process that can readily adapt to treatment within the lifetime of a patient. Although deep sequencing of primary and recurrent tumors, and liquid biopsy, have been used to track tumor evolution in vivo, there is still a need for models that can be manipulated in vitro to accurately and directly describe the detailed evolutionary trajectory of drug-resistance in cancer and identify new mechanisms of drug-resistance.

In the present study, we first established a set of 18 cell lines selected for sequential MTX-resistance of the human colon cancer cell HT-29, some of which carried HSRs or ecDNAs. We then investigated genome-wide copy number changes across all of these cell lines to understand the detailed and gradual landscape of changes in genome evolution under MTX selection, as well as the possible mechanisms and the driving force of MTX-resistance. This study thus provides a comprehensive and detailed picture of genomic dynamic changes and insights into the underlying mechanism of MTX drug-resistance development. Our work could also provide potential new targets for combating drug resistance in cancer treatment and suggest future directions of drug-resistance studies.

## Materials and methods

### MTX-resistant cell line establishment

The original HT29 cell line (S-0, stage 0) was purchased from the Type Culture Collection of the Chinese Academy of Sciences (Shanghai, China, http://www.cellbank.org.cn/). Initially, this cell line was cultured in Dulbecco’s modified Eagle’s medium (DMEM; GibcoBRL, Gaithersburg, MD, USA) supplemented with 1.0 × 10^−7 ^mol/L MTX (Pfizer (Perth) Pty, Bentley WA, Australia) to induce resistance. After cell growth was stable, the next concentration of MTX was added successively to induce drug resistance until the concentration of MTX was 6.0 × 10^−4^ mol/L. All the cell lines (S-1-S-18:1.0 × 10^−7^, 2.0 × 10^−7^, 4.0 × 10^−7^, 6.0 × 10^−7^, 8.0 × 10^−7^, 1.0 × 10^−6^, 2.0 × 10^−6^, 4.0 × 10^−6^, 6.0 × 10^−6^, 8.0 × 10^−6^, 1.0 × 10^−5^, 2.0 × 10^−5^, 4.0 × 10^−5^, 6.0 × 10^−5^, 8.0 × 10^−5^, 1.0 × 10^−4^, 3.0 × 10^−4^ and 6.0 × 10^−4 ^mol/L of MTX resistant cells) were cultured in the presence of 15% fetal calf serum (PAA Laboratories GmbH, Pasching, Austria). Human osteosarcoma cell line U2OS (Shanghai, China, http://www.cellbank.org.cn/) and its MTX-resistant cell lines U2OS e-6 and U2OS e-4 (the concentrations of MTX resistance were 1.0 × 10^−6^ mol/L and 1.0 × 10^−4^ mol/L, respectively), previously constructed by our research group, were also cultured in DMEM with 15% fetal calf serum. All cell lines were negative for Mycoplasma contamination.

### Characterization of the MTX-resistant cell lines

Cell viability was assessed using CellTiter 96® AQ_ueous_ One Solution (Promega, Madison, WI, USA). Cells (5000/well) were treated with different concentrations of MTX from 1.0 × 10^−9^ mol/L to 1.0 × 10^−2 ^mol/L for 72 h and incubated with 20 μl *3-*(4,5-dimethylthiazol-2-yl)-5-(3-carboxymethoxyphenyl)-2-(4-sulfophenyl)-2H-tetrazolium (MTS) for 3 h. Then optical density (OD) values of the MTS solution were measured using a microplate reader (Tecan, Grödig, Austria) at 492 nm wavelength. The values of the half-maximal inhibitory concentration (IC_50_) were calculated.

*DHFR* gene copy numbers were measured using real-time PCR performed with the LightCycler 480 system (Roche Applied Science, Mannheim, Germany). Genomic DNA was extracted using a QIAmp DNA Mini Kit (Qiagen, Düsseldorf, Germany) following the instructions from the manufacturer. The DNA primers used are listed in Table S10 with β-actin as control. The amplification steps of all these genes were performed for 45 cycles of 20 s at 95 °C, 20 s at 60 °C and 30 s at 72 °C.

Metaphase spreads from Colcemid (Sigma-Aldrich Co.LLC, Saint-Louis, MO, USA) arrested cells were prepared according to standard cytogenetic methods [15]. DNA from the BAC clones PR11-90A9 (BAC PAC Resources Center, Oakland, CA, USA) and/or RP11-91I22 (BAC PAC Resources Center, Oakland, USA) was extracted using a Genopure Plasmid Midi Kit (Roche Applied Science, Mannheim, Germany) and labeled with Green-dUTP and Red-dUTP, respectively, using the BioPrime DNA Labeling System Kit (Invitrogen, Carlsbad, CA, USA). Hybridization to metaphase spreads was as described in our previous study [15], and the slides were counterstained with DAPI. Images were obtained using a fluorescence microscope equipped with the MetaMorph Imaging System 7.7.0.0 (Molecular Devices Corporation, Sunnyvale, CA, USA).

### Genome-wide CNV analyses

Genomic DNA (>1.5 μg) was randomly segmented to an average of 350 bp and subjected to DNA library creation with the Illumina NGS DNA Library Construction Kit (Illumina, San Diego, CA, USA). Whole-genome sequencing data were generated by Novogene (Beijing, China) using PCR-based libraries and 150 base paired-end sequencing on the Illumina HiSeqX Ten platform. The mean sequence depth on the 19 cell lines was 32×, with mean coverage of the genome 98.94% (Table S2). Control-FREEC was applied to call CNVs from NGS data. Copy numbers of chromosomal segments are shown in Supplementary Material 1. A SNP array was also used to detect copy number changes. CNV calling from SNP array data (Figs. S1, S2 and Table S8,S9), and the comparison of CNVs from sequencing and SNP genotyping data are shown in Table S3. We counted the total amount of amplified (gained) DNA by adding together the length of all CNVs with copy number greater than 2, and the total amount of deleted (lost) DNA by adding the length of all CNVs with copy number less than 2 in each cell line. The net amount of DNA changes was calculated as the total gain minus the total loss. Lowess curve fitting was performed on the total DNA gain, loss and net change plotted against MTX-resistance level, and the Pearson correlation was calculated. All these analyses were performed using R (R-3.2.0, win-64).

### MTX-resistance-specific CNV analyses

CNVs called from sequencing data were used for this analysis. We identified the CNVs in each of the MTX-resistant HT29 cell lines (S1−S18) which differed from the original HT29 cell line (S0), requiring less than 50% overlap between them. We defined this set of CNVs as MTX-resistance-specific CNVs (MRS-CNVs). We also filtered out MRS-CNVs smaller than 10 kb (an arbitrary choice) (Figure S3).

As both genome-wide CNVs and MRS-CNVs were called per sample, we first merged all MRS-CNVs into a union call set containing the calls from all of the 18 cell lines. We kept cell-line-specific MRS-CNVs and shared ones with the same start and end coordinates as they were, but split the partially shared ones into cell-line-specific and completely shared ones. We then assigned genotypes for all of the union set across all of the 18 MTX-resistant cell lines. We finally weighted these CNVs (length of basic CNV × copy number of the basic CNV/ total length of all basic CNVs) for the hierarchical clustering and principal component analyses. Both analyses were performed with R (R-3.2.0, win-64).

Genes that completely overlapped with MRS-CNVs were obtained using GENCODE 19 annotation on Ensembl GRCh37. These genes were further annotated as contributing to MTX-resistance  or not, using the information from the Pharmacogenomics Knowledgebase (PharmGKB, https://www.pharmgkb.org/index.jsp) database.

The copy number and mRNA expression estimates for *DHFR*, *MSH3*, *ZFYVE16*, *FAM151B*, *ANKRD34B*, *SPZ1* and *MTRNR2L2* were validated using real-time PCR with the primers listed in Table S10, using β-actin as control. The PCR cycles were 20 s at 95 °C, 20 s at 60 °C and 30 s at 72 °C for 45 cycles. The protein expression level was measured using Western Blot with Beta Actin, Alpha Tubulin or GAPDH for normalization. The antibodies used and their sources are listed in Table S11.

### siRNA transfection

Cells were seeded into 6-well plates at a density of 2 × 10^4^ cells per well. Transfection was performed using 80 pmol of small interfering RNAs (siRNAs) (siDHFR, siMSH3, siZFYVE16 and siFAM151B) or control siRNA. jetPRIME reagent (PolyPlus Transfection, Strasbourg, France) was used for transfection following the manufacturer’s instructions. Transfection efficiency was assessed after 48 h. The sequences of siRNAs are listed in Table S12.

### Cell counting kit-8 (CCK-8) assay

To analyze the effects of DHFR, MSH3, ZFYVE16 and FAM151B on MTX resistance, cells transfected with target gene siRNAs and control siRNA were harvested and seeded into 96-well plates at a density of 3000 cells per well. The cells were then treated with MTX for 48 h. Following this incubation period, the reagent of CCK-8 (GLPBIO, Montclair, CA, USA) was added to each well, and the plates were incubated for 2 h. Absorbance at 450 nm was measured to calculate the IC_50_ value for MTX.

### Statistics

Statistical analysis was performed using the R program (v3.2.3). Differences between different groups (sample size = 3) were analyzed using a two-tailed Student′s *t*-test or one-way ANOVA analysis. The correlation coefficient was calculated using the Pearson method with R built-in function “cor()”. A Lowess curve was fitted with the R built-in function “lowess()”. Measurement data were presented as mean ± standard deviation (SD) of three independent experiments. Prior to the analysis, a normality test and a homogeneity of variance test were performed on all collected data. Significance was indicated by asterisks: *P < 0.05; **P < 0.01; ***P < 0.001.

## Results

### Establishment of MTX-resistant HT-29 colon cancer cell lines

**Fig. 1:**
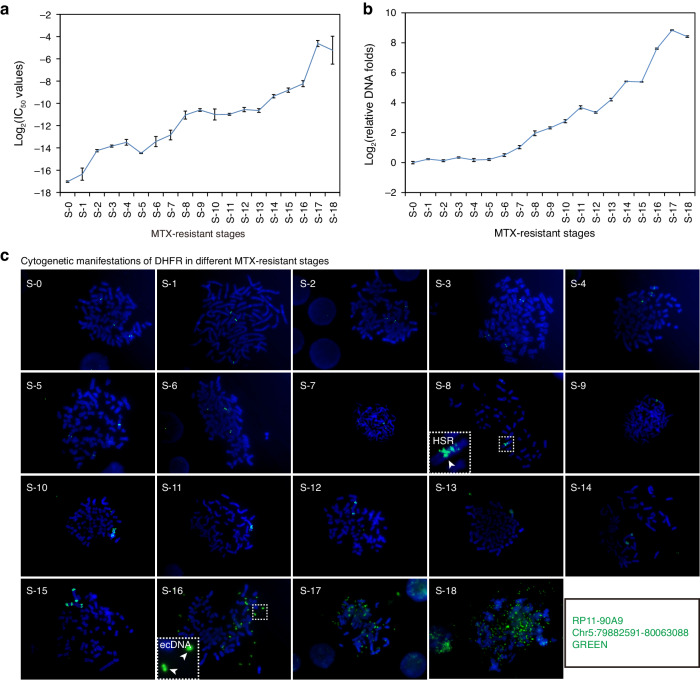
Characteristics of the MTX-resistant HT29 cell lines. **a** The IC_50_ of HT29 and HT29 MTX-resistant cell lines. The x-axis shows the level of MTX-resistance and the y-axis shows the log_2_ of the IC_50_ value. **b** *DHFR* gene copy numbers of HT29 and HT29 MTX-resistant cell lines measured by real-time PCR. The y-axis is the log_2_ of the copy number relative to S0, and the x-axis is the level of MTX-resistance. Data in (**a**, **b**) are present as mean ± standard deviations. **c** Cytogenetic manifestations of *DHFR* in different MTX-resistant cell lines. The RP11-90A9 probe containing *DHFR* is labeled green. The 18 MTX-resistant cell lines can be classified into 3 groups (S-1 ~ S-7, S-8 ~ S-15 and S-16 ~ S-18), whose cytogenetical manifestations were pre-amplification, HSR and ecDNA, respectively.

We successfully established a series of 18 MTX-resistant cell lines from the HT-29 colon cancer cell by sequentially increasing MTX concentrations. As expected, the IC_50_ values increase as the concentrations of MTX used for selection increase (Fig. 1a and Table S1), while the copy number of the *DHFR* gene also increases, as shown by both real-time PCR and metaphase FISH (Fig. 1b, c). Metaphase FISH also shows that HSRs and ecDNAs arise at stage S-8 and S-16, respectively. So we classified the 18 MTX-resistant HT29 cell lines into three groups based on the *DHFR* amplification forms: pre-amplification group, S-1 to S-7 before *DHFR* amplified; HSR group, S-8 to S-15 with moderate *DHFR* amplification; and ecDNA group, S-16 to S-18 where the *DHFR* gene is highly amplified (Fig. 1c).

### Amplifications played important roles in the evolution of MTX-resistance

**Fig. 2:**
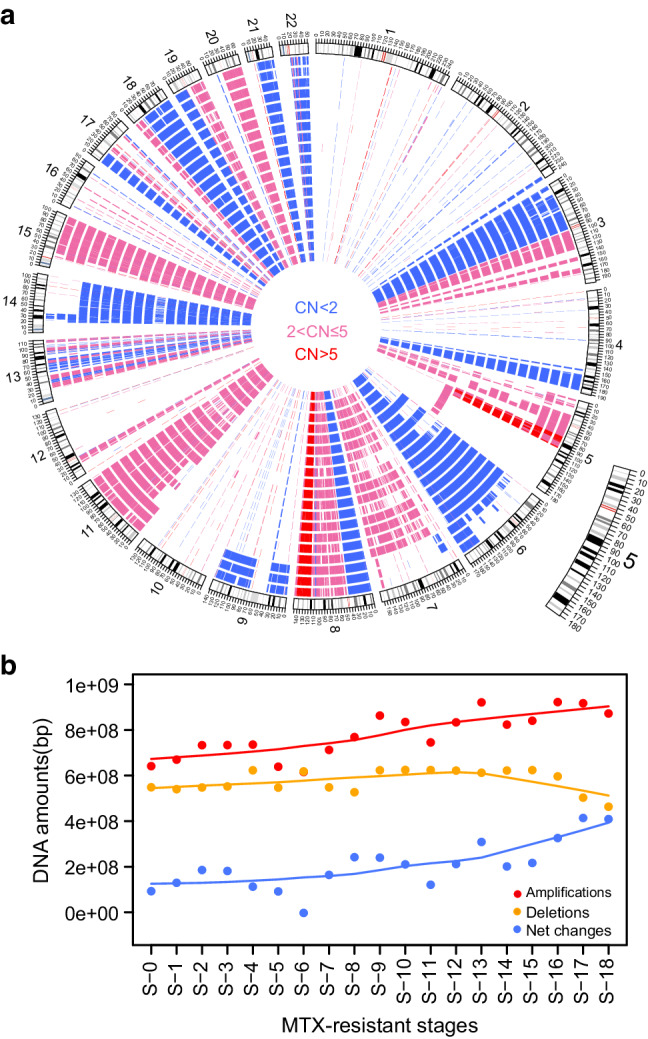
Genome-wide copy number variation changes in HT29 and its MTX-resistant cell lines. **a** Circos plot of genome-wide CNVs called from the whole genome sequencing data in HT29 and its MTX-resistant cell lines. Amplification of 2 < CN ≤ 5 is shown in pink, CN > 5 is in red, while deletions are in blue. The cell lines S-0, S-1, S-2… and S-18 are displayed from the inner to the outer circles. **b** The amount of nuclear DNA changes in the different stages of MTX-resistant cell lines. The net change curve is in orange, amplification in red while the deletions are in blue. The curve is fitted using the Lowess method.

The quality control on the CNV call set from sequencing data is shown in Table S2 and Table S3. We found many copy number changes, both amplifications and deletions, across the genome which were shared by all of the 19 cell lines: for example, amplifications on chromosomes 3, 8, 11 15, 18, 19, and 20, and deletions on chromosomes 3, 4, 8, 13, 17, 18, 19, 21 and 22 (Fig. S4). These CNVs are likely part of the genomic architecture of the original cell line. We also found changes that are not shared, for example, the amplification on chromosome 5 and the deletions on chromosome 9 and 14 (Fig. 2a). In addition, we found that the total numbers of DNA changes across all cell lines are significantly correlated with the level of MTX resistance (Pearson correlation coefficient *R* = 0.51, *P* = 8.037 × 10^−4^) and the amplifications are the main driver for this correlation (Fig. 2b). However, we did not find significant changes of mitochondrial DNA copy number in the different stages of the cell lines (Table S4). These observations suggest that amplification is very important for allowing the cell line to survive under MTX selection.

### MTX-resistance-specific amplifications are the main drivers during evolution

**Fig. 3:**
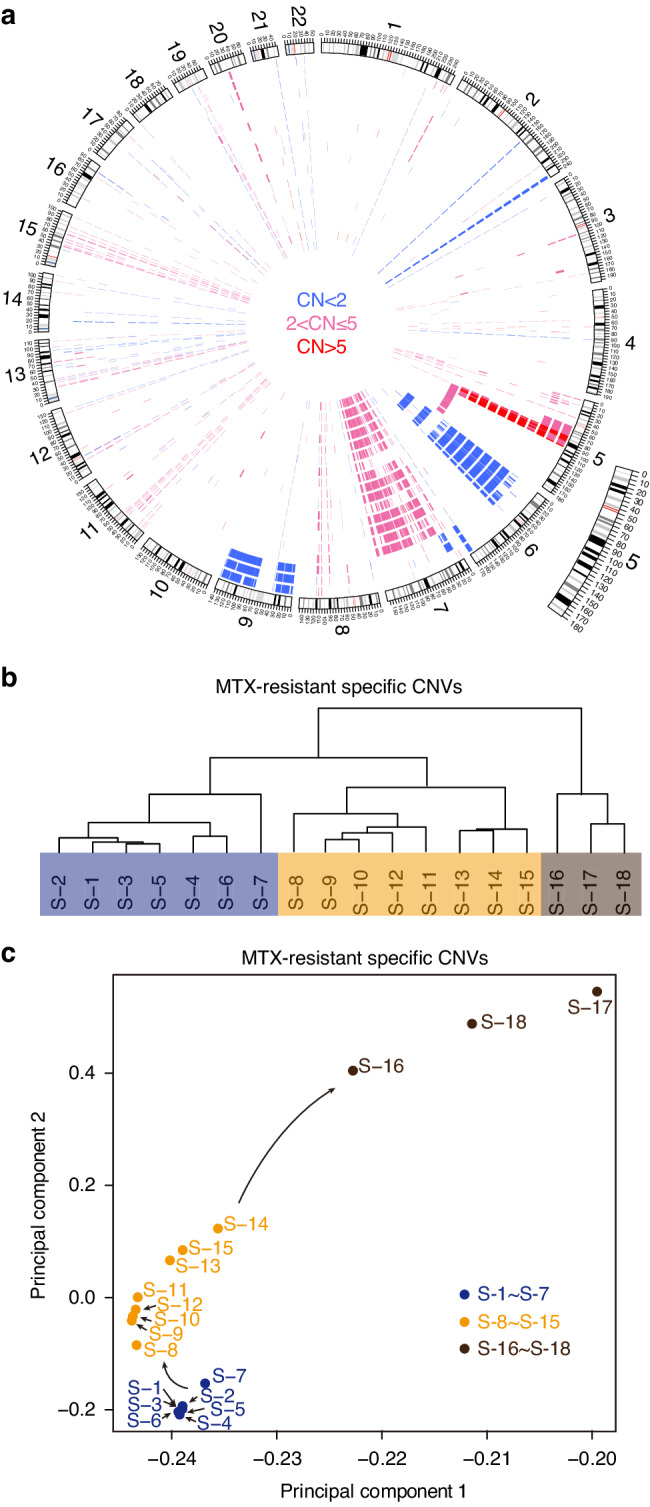
Analyses of CNVs specific to each MTX-resistant cell line (MRS-CNVs). **a** Circos plot of the MRS-CNVs of each MTX-resistant cell line, compared with the original HT29 cell line. All shared CNVs between HT29 and each of the 18 HT29 MTX-resistant cell line were excluded. Amplification of 2 < CN ≤ 5 is in pink, CN > 5 in red, and deletions in blue. The cell lines S-1, S-2… and S-18 are ordered from inside to outside. **b**, **c** Hierarchical clustering and PCA of MRS-CNVs in the different MTX-resistant cell lines. The 18 cell lines are clustered into 3 groups; S-1 to S-7, S-8 to S-15 and S-16 to S-18, respectively.

To understand the specific CNV changes in each stage of the MTX resistance, MTX-resistance-specific CNVs (MRS-CNVs) in each MTX-resistant cell line were identified (Figs. 3a, S4 and S5). Table S5 shows both MRS-amplifications and MRS-deletions specific to different stages of MTX-resistance. All of the cell lines within the pre-amplification, HSR and ecDNA groups cluster according to their group in a hierarchical clustering analysis with the copy numbers weighted by the length of the MRS-CNVs, and the pre-amplification group clusters with the HSR group first, then with the ecDNA group (Fig. 3b), suggesting more MRS-CNV changes in the ecDNA group. Similar results were also seen in a principal component analysis (PCA) (Fig. 3c). When we carried out further hierarchical clustering and PCA using the MRS-amplifications and MRS-deletions separately, we found that the cell lines grouped into pre-amplification, HSR and ecDNA groups with the MRS-amplifications (Fig. S6a, 6c) but not with MRS-deletions (Fig. S6b, 6d), which further suggests the MRS-amplifications are main drivers for the MTX-resistance while the MRS-deletions may be by-products resulting from the genomic instability. In addition, we also examined the ploidy of HT29 MTX-resistant cells at different groups using FISH, the result showed that the MRS-amplification was not caused by a genome doubling event (Fig. S7).

### Candidate biological mechanisms in the different MTX-resistant stages

**Fig. 4:**
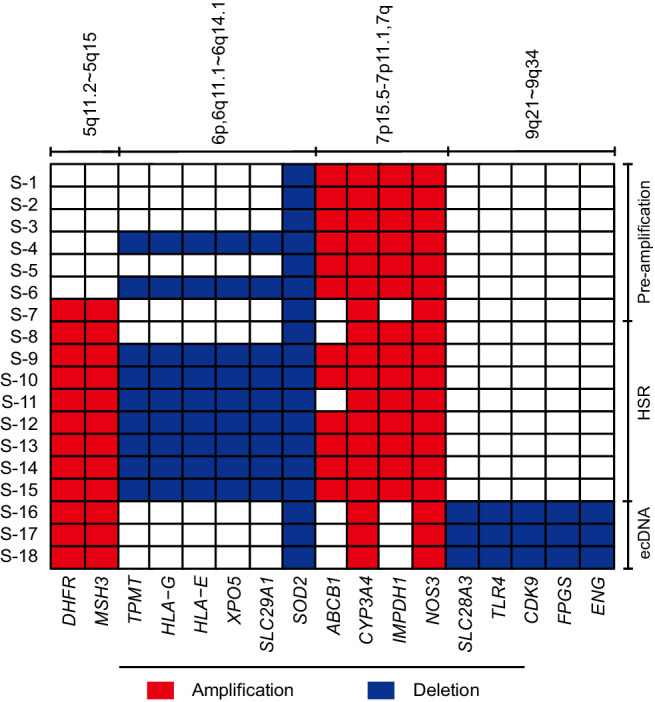
Gene annotation within the regions of MRS-CNVs shared by each group or the groups of MTX-resistant cell lines. The locations and types of MRS-CNVs are shown on the top of the diagram, and the different groups are highlighted by different colors. The gene names within each MRS-CNVs annotated using information from the Pharmacogenomics Knowledgebase (https://www.pharmgkb.org), are shown on the bottom of the diagram.

The genes that overlap with MRS-CNVs and their annotations are shown in Fig. 4, Tables S5 and S6. Three known MTX-resistance related genes were found in all stages, with deletion of *SOD2* and amplifications of *CYP3A4* and *NOS3*. *ABCB1* and *IMPDH1* were observed amplified at all pre-amplification and HSR stages. Some other MTX-resistance related genes, such as *TPMT*, *HLA-G*, *HLA-E, XPO5* and *SLC29A1* were seen as deletion CNVs in some of the pre-amplification and most of the HSR stages, while *SLC28A3*, *TLR4*, *CDK9*, *FPGS* and *ENG* showed as deletions in the ecDNA stages. *DHFR* and *MSH3*, another two known MTX-resistance related genes were amplified at the last level of the pre-amplification stage and throughout the HSR and ecDNA stages. As the same time, we found that these two genes carried different SNPs at these stages compared with the unamplified copies of *DHFR* and *MSH3* (Supplementary Material 2).

### Major driver genes in the evolution of MTX-resistance

**Fig. 5:**
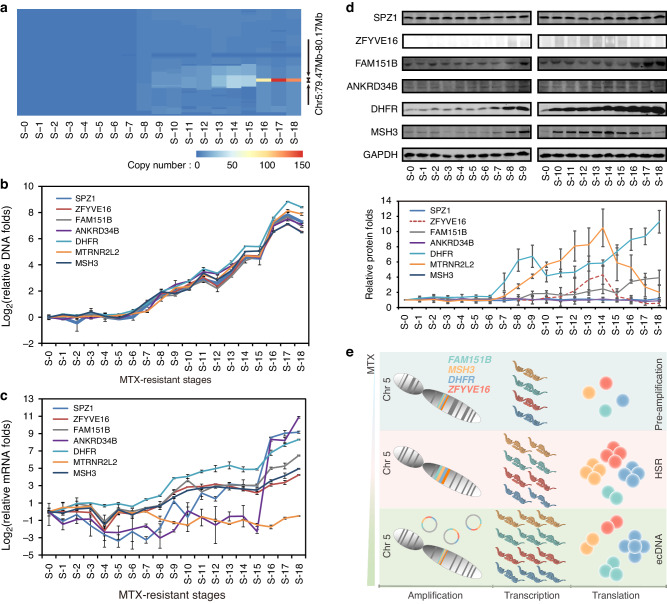
Potential driver genes during MTX-resistance cell line development. **a** Heat map of copy numbers of the highly amplified MRS-CNV located on chromosome 5: 79474000−80170000 and surrounding region. The copy number of this region increases with MTX concentration (Pearson correlation coefficient = 0.86, *P* < 0.05), especially significant in stages of S-16 to S-18. **b** The copy number changes of 7 protein-coding genes (*MSH3, DHFR, ANKRD34B, SPZ1, FAM151B, ZFYVE16 and MTRNR2L2*) within chromosome 5: 79474000−80170000 measured by real-time PCR. This further validated the observation from whole genome sequencing data. **c** The mRNA expression level of the 7 amplified protein-coding genes. All of the 7 genes except *MTRNR2L2* showed similar changes. **d** The protein level of the 6 protein-coding genes with increased expression by Western Blot. Only the level of FAM151B, ZFYVE16, DHFR and MSH3 showed a gradual increase as MTX-resistance level increased (We could not perform gray-scale analysis of ZFYVE16 because its expression was not detectable in many cell lines. Therefore, we simulated the expression curve with dashed line across detectable cell lines). Data in (**b**, **c**, **d**) are present as mean ± standard deviations. **e** Evolutionary model of high expressed genes on ecDNAs at different phases of MTX resistance in cancer cells.

**Fig. 6:**
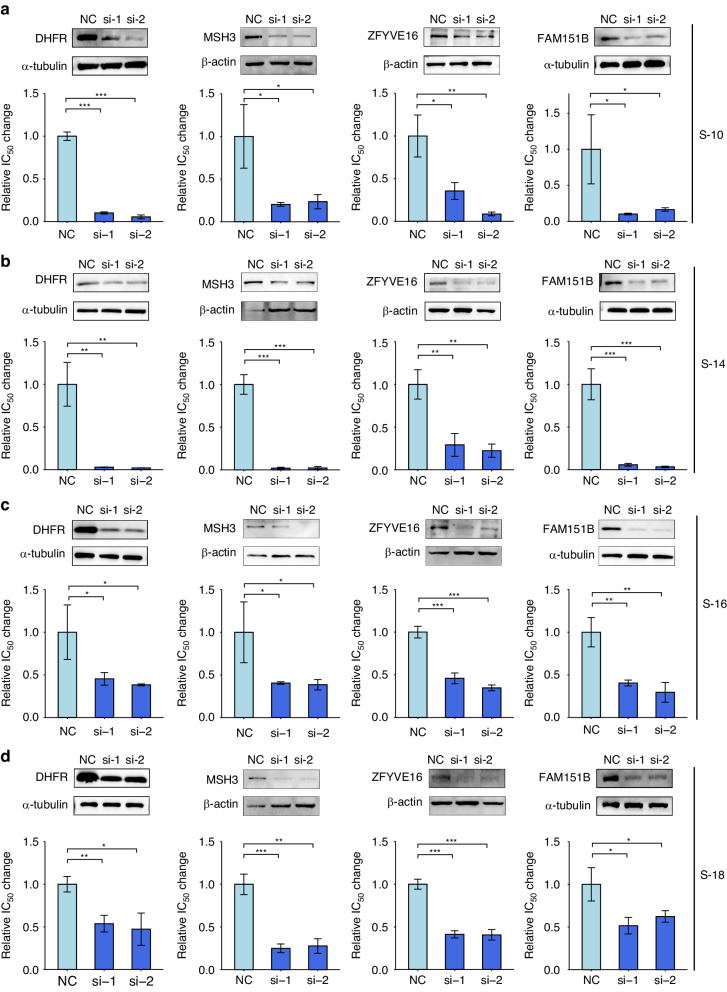
Effect of potential driver genes on the acquisition of MTX resistance. The relative IC_50_ changes of HSR (**a**:S-10, **b**: S-14) and ecDNA (**c**:S-16, **d**:S-18) phase cells, after inhibiting the expression of *DHFR, MSH3*, *ZFYVE16* and *FAM151B*, respectively. siRNAs were used to inhibit genes expression, and the interference effect was detected by Western Blot, while the IC_50_ values of cells was detected by the CCK8 method. Data in (**a**−**d**) are presented as mean ± standard deviations and analyzed by Student’s *t*-test (*n* = 3 independent experiments; ***P < 0.001, **P < 0.01, *P < 0.05).

The amplification of the chr5:79,474,000−80,170,000 region which includes both *DHFR* and *MSH3* plays an important role in the evolutionary process, as its copy numbers are highly correlated with the concentration of MTX-resistance (Pearson correlation coefficient *R* = 0.86, *P* = 4.14 × 10^−6^) (Fig. 5a). In addition, we found that the 18 cell lines could not be separated into the three groups any more if we excluded this CNV in both the hierarchical clustering and PCA analyses (Fig. S6e, 6f). Another five genes, *ANKRD34B*, *SPZ1*, *FAM151B*, *ZFYVE16* and *MTRNR2L2* were also amplified in this region (Fig. 5b and Table S7). The expression of all except *MTRNR2L2* increased in both HSR and ecDNA stages, and increased more significantly in ecDNA stages, especially *ANKRD34B* and *SPZ1*. Only DHFR, MSH3, ZFYVE16 and FAM151B showed protein level changes in the different stages. In the ecDNA stages, the expression of DHFR was significantly higher than that in the HSR stages, and the expression of FAM151B was slightly higher than in the HSR stages. The expression of MSH3 and ZFYVE16 showed a similar trend, which increased in most HSR stages, decreased suddenly in the late stages of HSR, and decreased more sharply in the ecDNA stages (Fig. 5c–e).This may be related to the instability of ecDNAs, but it is more likely due to the expression of these two proteins from the ecDNAs being disrupted (Fig. S8). We also found that *DHFR*, *MSH3* and *ZFYVE16* were highly expressed in almost all the MTX resistance cells randomly selected in the GEO database, while no expression results were detected for *FAM151B* (Fig. S9). Finally, we inhibited the expression of *DHFR*, *MSH3*, *ZFYVE16* and *FAM151B* at the HSR and ecDNA stages, respectively, and found that the relative IC_50_ changes of cells against MTX was significantly reduced, indicating that the amplification of these four genes played an important role in the resistance of tumor cells to MTX (Fig. 6). We also confirmed this finding in MTX-resistant U2OS cells (Fig. S10). These suggest that *DHFR* amplification is the main driver for MTX-resistance from this genomic region of chr5:79,474,000-80,170,000 in both HSR and ecDNA phases, although *MSH3, ZFYVE16* and *FAM151B* could also contribute.

## Discussion

Cancer is a genomic disease and the dynamic mechanisms of drug resistance in cancer cells remain incompletely understood. It has long been known that *DHFR* gene amplification is a key cause for MTX resistance in cancer patients. However, our results suggest that MTX resistance is far more complicated.

MTX has been reported as an inducer of single and double strand breaks of DNA, and DSB repair systems were found to be highly expressed in MTX-resistant cancer cells in our previous studies [15, 16]. Thus, we hypothesized that genomic instability may occur during the evolution of MTX-resistance, which was confirmed by the specific amplification on chromosome 5 and specific deletions on chromosomes 9 and 14. Amplification events were found to dominate in the evolution of MTX-resistance by analysing the correlation between DNA contents of CNVs and MTX-resistance, which indicates that amplification can be seen as a way to extend the range of gene expression to handle extreme conditions [2]. Meanwhile, some deletions in the genome were also detected, which may be suspected to be compensations for balancing the contents size of the cell genome. Further, the evolutionary process of MTX-resistance in the cell lines we studied can be divided into three phases according to the clustering and principal component analyses of MRS-CNVs, which are consistent with the cytogenetic manifestations of *DHFR* (pre-amplification, HSR and ecDNA) in the evolutionary model. We believe that significant genomic instability, especially amplification, underlies the development of MTX resistance.

By comparing the genes in the MRS-CNV regions with the known MTX-resistance associated genes, different sets of genes were found involved in the different phases of resistance. *CYP3A4* is involved in the metabolism of many anticancer drugs, and its expression may be induced by MTX [17]. *NOS3* expression can be affected by *DHFR* regulating the ratio of BH4 to BH2 [18, 19], therefore, its expression is related to the increase of MTX resistance [20]. The amplification of *CYP3A4* and *NOS3* throughout the course of MTX resistance was discovered to be associated with MTX resistance in our study for the first time. *ABCB1* encodes multidrug resistance 1 (MDR1), which can provide resistance to a very broad range of cytotoxic and targeted chemotherapy agents [21–23]. *IMPDH1* catalyzes a key step in guanine nucleotide biosynthesis [24, 25], as a homotetramer regulating cell growth. We also found for the first time that *ABCB1* and *IMPDH1* amplifications played roles in the earlier stages of MTX resistance. Our results suggest that amplification induced by genomic changes takes place earlier than the well-known *DHFR* amplification, which adds further to our understanding of the mechanism of MTX resistance. *DHFR* amplification is a well-known MTX-resistance mechanism, and the co-amplification of *DHFR* and *MSH3* is usually observed in MTX-resistant cancer cells [26–28]. However, we now have found that the amplification of these two genes only appeared at the later stages of MTX-resistance, which suggests that *DHFR* and *MSH3* amplification could be a selected mechanism of cancer cells to adapt to higher concentrations of MTX. The MRS-CNV containing *DHFR* and *MSH3*, chr 5:79,474,000−80,170,000, whose copy number was highly correlated with MTX-resistance concentrations, and the different stages of the cell lines would no longer aggregate into three phases without this MRS-CNV, which also underlines the significance of chr 5:79,474,000−80,170,000: it may drive MTX resistance through HSR formation at earlier stages and ecDNA at later stages.

Amplification of oncogenes or proliferation-related genes usually plays a central role in tumorigenesis by providing cancer cells with selective growth advantages through overexpression of amplified genes [29]. Alt et al. initially discovered that tumor cells can develop MTX-resistance through amplification of the *DHFR* gene [30] either on an “unstable” form of gene amplification (ecDNA) or a “stable” form (HSR) [31, 32]. Using our cell line model, we further discover that ecDNA can provide more elevated copy numbers than HSR due to the lack of a centromere leading to unequal segregation at cell division, suggesting a more pivotal role of ecDNA in adapting to selective pressures from cancer therapy. Previous studies have shown that ecDNA may dynamically relocate to chromosomal HSR [5, 33]. Other studies also found that HSR may promote the formation of ecDNA [34, 35]. In our evolutionary model, we confirm for the first time that ecDNA derives from HSR with key structural features under higher MTX stress. Thus, these findings support the idea that *DHFR* and *MSH3* can effectively promote MTX-resistance of cancer cells in the forms of HSR and ecDNA, especially ecDNA, which can adapt to the pressure of higher MTX resistance as a driver of selection.

In addition to *DHFR* and *MSH3*, another five genes (*ANKRD34B*, *SPZ1*, *FAM151B*, *ZFYVE16* and *MTRNR2L2*) were located on chr5: 79,474,000−80,170,000, and it is important to understand their roles in MTX resistance. We found that in both HSR and ecDNA phases, all 7 amplified genes were highly expressed except *MTRNR2L2*, suggesting that not all the genes located in HSR/ecDNA, especially ecDNA, may play a role. Further, the mRNA expression of these 6 genes, especially *ANKRD34B* and *SPZ1* increased more significantly in ecDNA stages than in HSR stages. We hypothesize that distal DNA elements may be brought into proximity, enabling chromatin interaction and potentially forming new gene regulatory circuits, due to the altered 3D topology of ecDNA [36, 37]. And ecDNA may also play a pivotal role in reorganizing transcriptional control by enhancer hijacking [36, 38]. At the protein expression level, only DHFR and FAM151B increased steadily throughout the MTX resistance process, especially DHFR during the ecDNA stages, while the expressions of MSH3 and ZFYVE16 during the ecDNA stages decreased relative to HSR stages. Here, the decreased expression of MSH3 and ZFYVE16 are unexpected and interesting, suggesting that co-amplification of genes in ecDNA does not necessarily lead to their co-expression as common trends and there may be other posttranscriptional mechanisms for differential regulation of genes on HSR and ecDNA, respectively. Our study directly demonstrates that high expression of DHFR due to genomic alterations (HSR and ecDNA, especially ecDNA) is the major driving force behind MTX resistance in cancer cells, whereas previous reports on the important role of ecDNA in tumor evolution have mostly lacked direct evidence. For the amplified genes in ecDNA other than *DHFR*, some may primarily reflect genomic proximity to a few driver genes and not necessarily lead to meaningful functional enrichment, although some of them may be controlled by 3D transcriptional regulation. Other genes also play different roles in different stages of MTX resistance, among which *ZFYVE16* and *FAM151B* were first found to contribute to MTX resistance. Inhibiting the expression of *DHFR*, *MSH3*, *ZFYVE16* and *FAM151B* can reduce the IC_50_ values of the cells against MTX, which confirms the important roles of these genes in MTX resistance, and we have also confirmed this finding in other MTX-resistant cells. Although the expression and amplification trend of these four genes in the process of MTX resistance are not completely consistent, they still play important roles in different phases of MTX resistance (HSR and ecDNA). From our results, the expression of amplified genes on ecDNA is inconsistent, and there may be complex regulatory mechanisms. Previous studies have shown that some drugs can eliminate ecDNAs and reverse the malignant or drug-resistant phenotypes [39, 40]. Also, our group has shown that the inhibition of the NHEJ and HR pathways may promote the excretion of ecDNAs in MTX-resistant cells and thus reverse drug resistance [15, 16]. Therefore, targeting ecDNAs and eliminating *DHFR* and other genes may be a promising new direction for cancer resistance to MTX therapy.

## Conclusions

Our results demonstrated that MTX-resistance in cancers is a complex and constantly changing process, and different genomic changes were shown at different phases which suggested different drug resistance driving mechanisms. Amplification of *CYP3A4*, *NOS3*, *ABCB1* and *IMPDH1* may be critically important before the *DHFR* amplification, which adds to our further understanding of the MTX resistance mechanism. EcDNAs are involved in the strong amplification and expression of *DHFR* and other genes, suggesting that ecDNAs play a key role in driving the evolution of MTX resistance. Thus, this work provides in-depth insight into the detailed dynamics of the genome changes during the development of drug resistance and hints at the potential implications for targeting ecDNAs and personalized therapies for cancer resistance.

### Supplementary information

Supplementray material_1

Supplementray material_2

Supplementray material_3

Supplementaryfiles

## Data Availability

All sequence data reported here have been deposited in NGDC, GSA database (https://bigd.big.ac.cn/gsa/) under the Accession Number of PRJCA017853. The data in the study are available on request from the corresponding author.
