# Supplementary material for: Dynamic genomic changes in methotrexate-resistant human cancer cell lines beyond DHFR amplification suggest potential new targets for preventing drug resistance
Source: Br J Cancer. 2024 Apr 9;130(11):1819–27. doi: 10.1038/s41416-024-02664-0 (PMC11130306; doi:10.1038/s41416-024-02664-0)
Supplement: Supplementary file 4 — Supplementaryfiles [file 41416_2024_2664_MOESM4_ESM.docx]

Supplementary files

Supplementary Figures


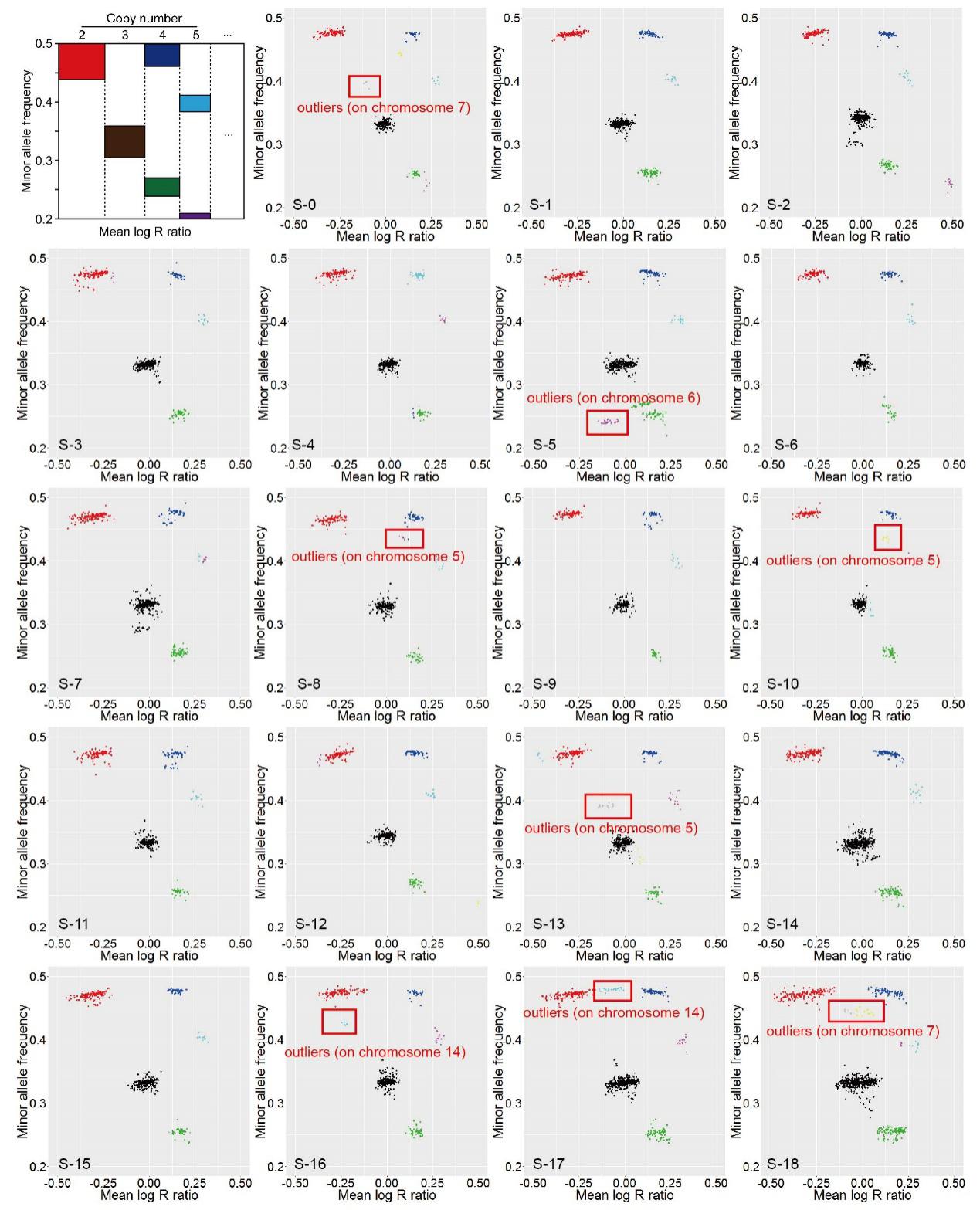


**Figure S1.** Distribution of mean log-R ratio (mLRR) and minor allele frequency (mAF) of segments with copy numbers of 2 to 5 in HT29 and 18 HT29 MTX-resistant cell lines.

Normalized log-R ratio (LRR) and B allele frequency (BAF, ranges 0.0 to1.0) of each probe from Illumina Human Omni 2.5-8 array (Illumina, San Diego, CA, USA) in each cell line were obtained as described below. Segmentation of LRR was carried out using the suggested parameters of aAlpha=0.8 and T=8^1,2^ and MinSegLen of 5 to minimize the false discovery rate (FDR), then the mean LRR (mLRR) of each segment was generated using the gada package in the R program (R-2.15.3, win-64). BAF of the probes within a segment were symmetrically distributed around 0.5, so the minor allele frequency of each probe was defined as 0.5 - abs(0.5-BAF). It ranges from 0 to ~0.5, and was used for further analysis, while the mAF of each segment was the median mAF of all non-loss of heterozygosity (non-LOH) probes (mAF>0.1; mAF less than 0.1 was treated as 0).

Based on previous studies^3,4^, mLRR and mAF of segments with different copy numbers have characteristic distributions, and the theoretical mLRR and mAF distributions of segments with copy numbers 2 to 5 are shown in top left corner of the Supplementary Figure S1. Clustering analysis using mLRR and mAF of non-LOH segments in 19 cell lines was done using the density-based spatial clustering of applications with noise (DBSCAN) via the fpc package in R, with parameter settings eps=0.021 and MinPts=4.

The normal copy number ploidy of a cell usually has a mLRR value of around 0, but the value was 3 for our dataset as the cell lines used in this study were triploid. Red boxes indicate the outliers that can’t be defined within standard copy number groups, and their corresponding segments are mainly concentrated on chromosomes 5, 6, 7 and 14 in all of the HT29 and HT29 MTX-resistant cell lines.


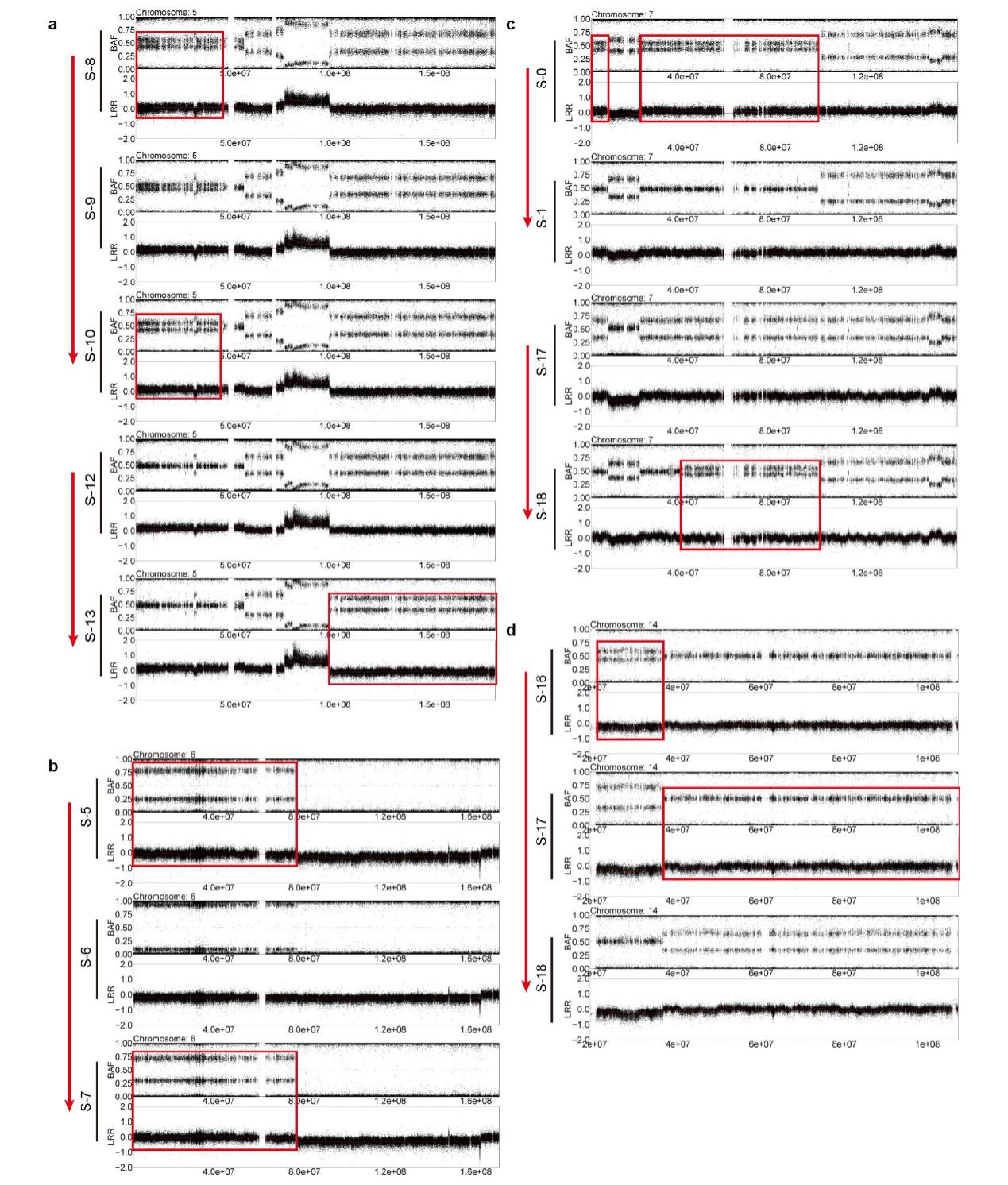


**Figure S2.** BAF and LRR of four example chromosomes.

These chromosomes 5 (a), 6 (b), 7 (c) and 14 (d) carry segments which are outliers, marked in red boxes in Supplementary Figure 1. Only subsets of the cell lines where the BAF changes, and their adjacent MTX-resistance stages are shown.


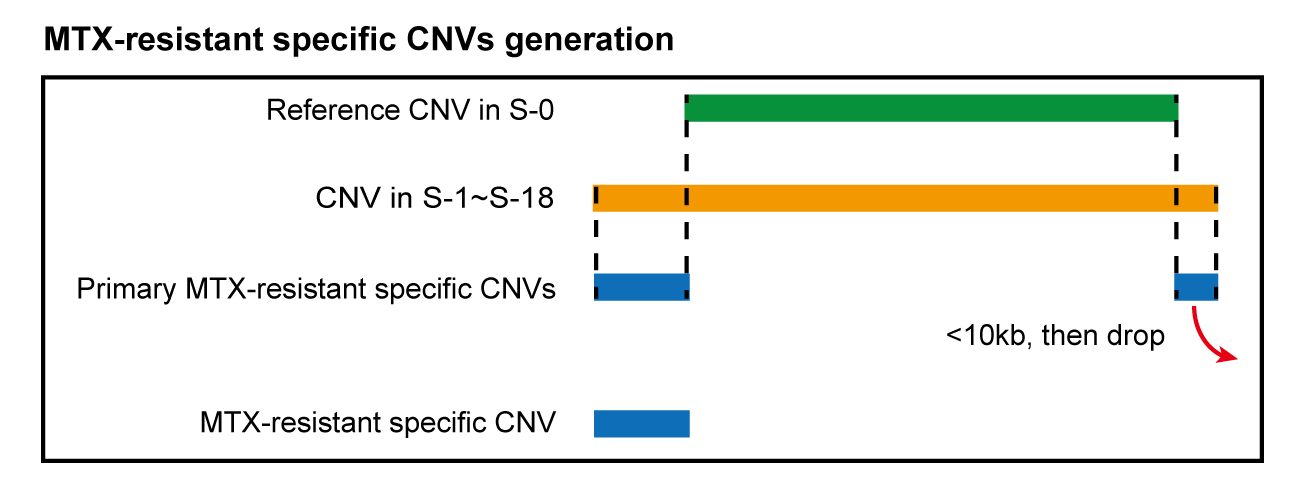


**Figure S3.** Protocol for definition of MTX-resistance-specific CNVs (MRS-CNVs). We filtered out shared CNVs between HT29 (green) and any HT29 MTX-resistant cell line (orange) to identify MTX-resistance-specific CNVs (blue) based on the characteristics of our own data (allowing large CNVs to be as long as a whole p/q arm). Since CNV breakpoints may not be detected exactly, this filtering procedure may generate artifactual small primary MRS-CNVs. We therefore dropped small primary MRS-CNVs less than 10 kb in size.


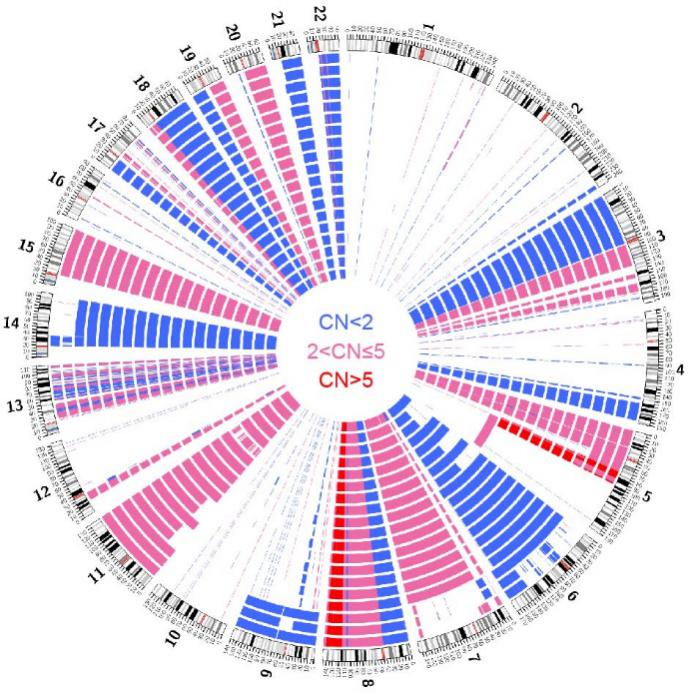


**Figure S4.** Circos plot of CNVs in HT29 and HT29 MTX-resistant cell lines from SNP intensity data. Highly amplified segments with CN greater than 5 are shown in red, ones with CN 3 to 5 in pink, and deletions with CN less than 2 in blue. The S-0, S-1, S-2 ... S-18 cell lines are shown in order from the inner to the outer circles. The normal ploidy of NGS data sets is 2, so the final copy number of the SNP chip data set was standardized by multiplying by 2/3, and the closest integer as the final result.


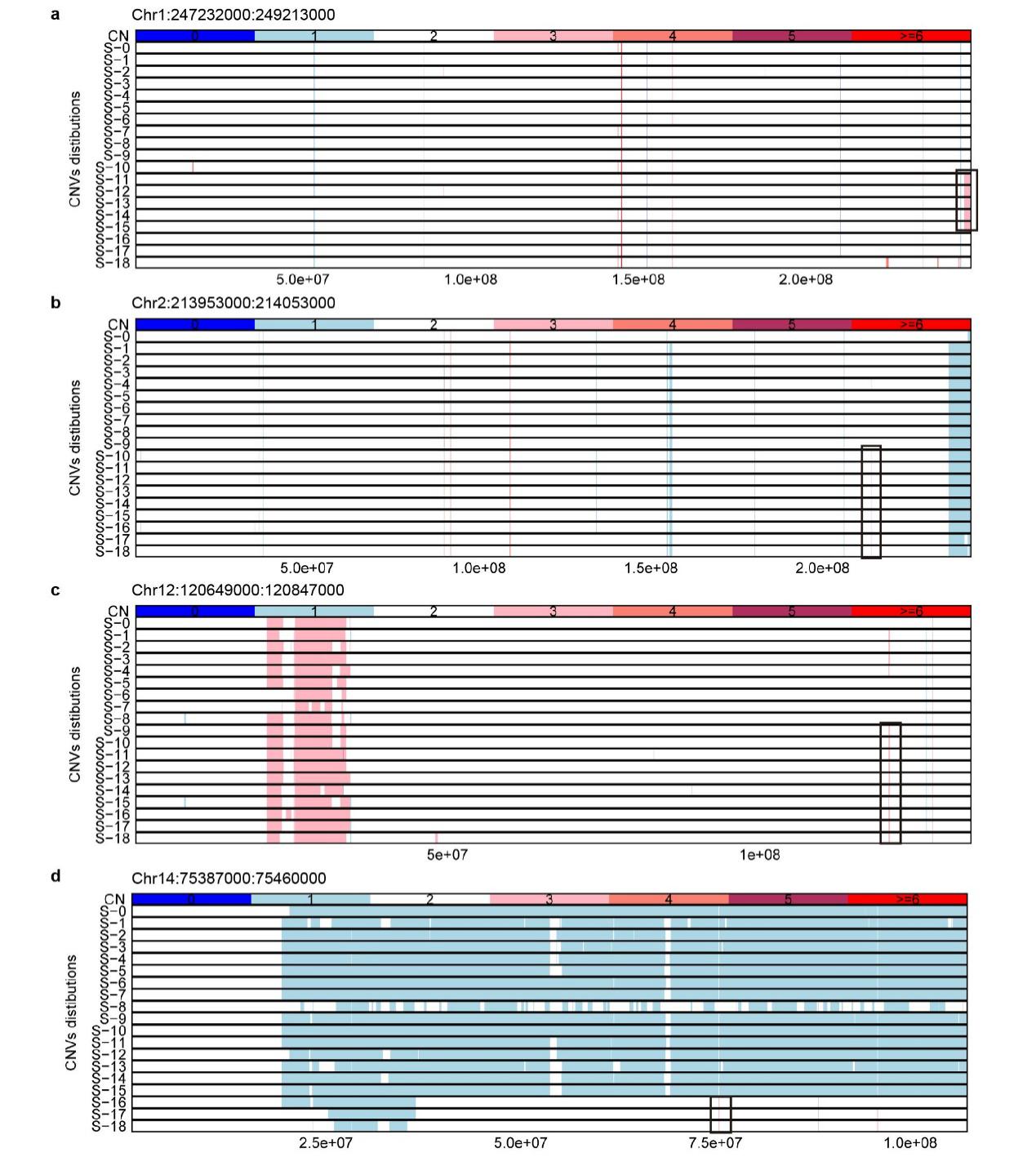


**Figure S5.** CNVs on four example chromosomes containing MRS-CNVs in HT29 MTX-resistant cell lines. Each line represents the cell line labelled on the left hand side, and the x-axis shows the chromosome coordinate. The amplified CNVs are in red and the deleted ones are in blue. MRS-CNVs are defined as those which are not in the original cell lines, but present continuously in several cell lines; the black boxes show examples.

1. MRS-CNVs on chr1:247232000:249213000.
2. MRS-CNVs on chr2:213953000:214053000.
3. MRS-CNVs on chr12:120649000:120847000.
4. MRS-CNVs on chr14:75387000:75460000.


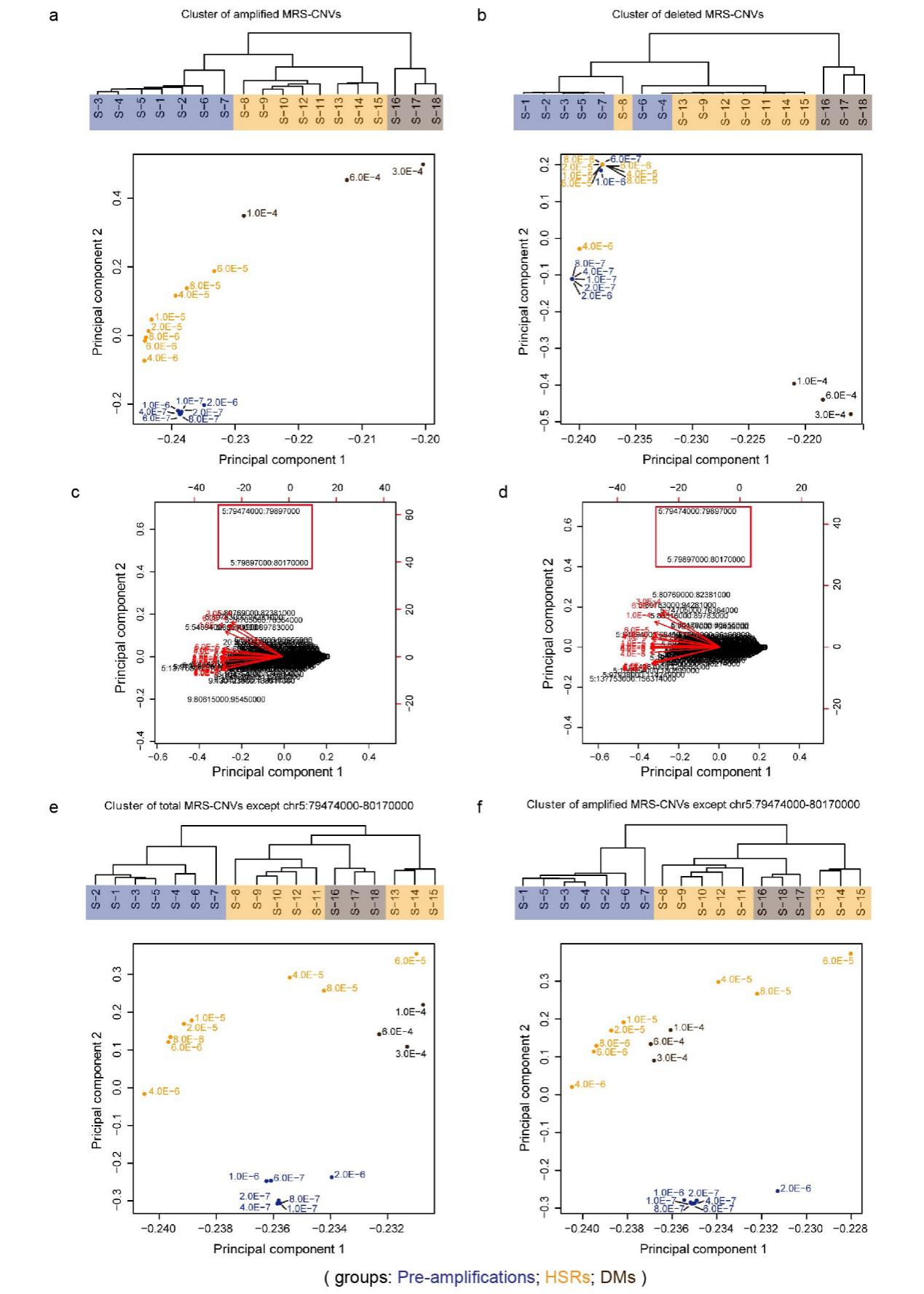


**Figure S6.** Genetic changes in the MTX-resistant cell lines from MRS-CNVs.

a. Hierarchical clustering and PCA analysis of amplified MRS-CNVs. The 18 MTX-resistant cell lines are clustered into 3 groups, similar to the groups using all MRS-CNVs.

b. Hierarchical clustering and PCA analysis of deletion MRS-CNVs. The clustering of 18 MTX-resistant cell lines is different from that using all MRS-CNVs.

c, d. Amplification of chr5: 79474000-80170000 might play an important role in MTX resistance. Based on the plot of MRS-CNVs at direction of component 1 and 2 using whole CNVs (c) and amplifications (d) respectively, chr5: 79474000-80170000 was highly amplified compared with other fragments, which implied that this segment might be under strong selection.

e, f. Hierarchical clustering and PCA analysis using all amplified MRS-CNVs (e) or except those from chr5: 7,9474,000-80,170,000 (f). The clustering of 18 MTX-resistant cell lines is different from that using all MRS-CNVs, or all amplified MRS-CNVs.


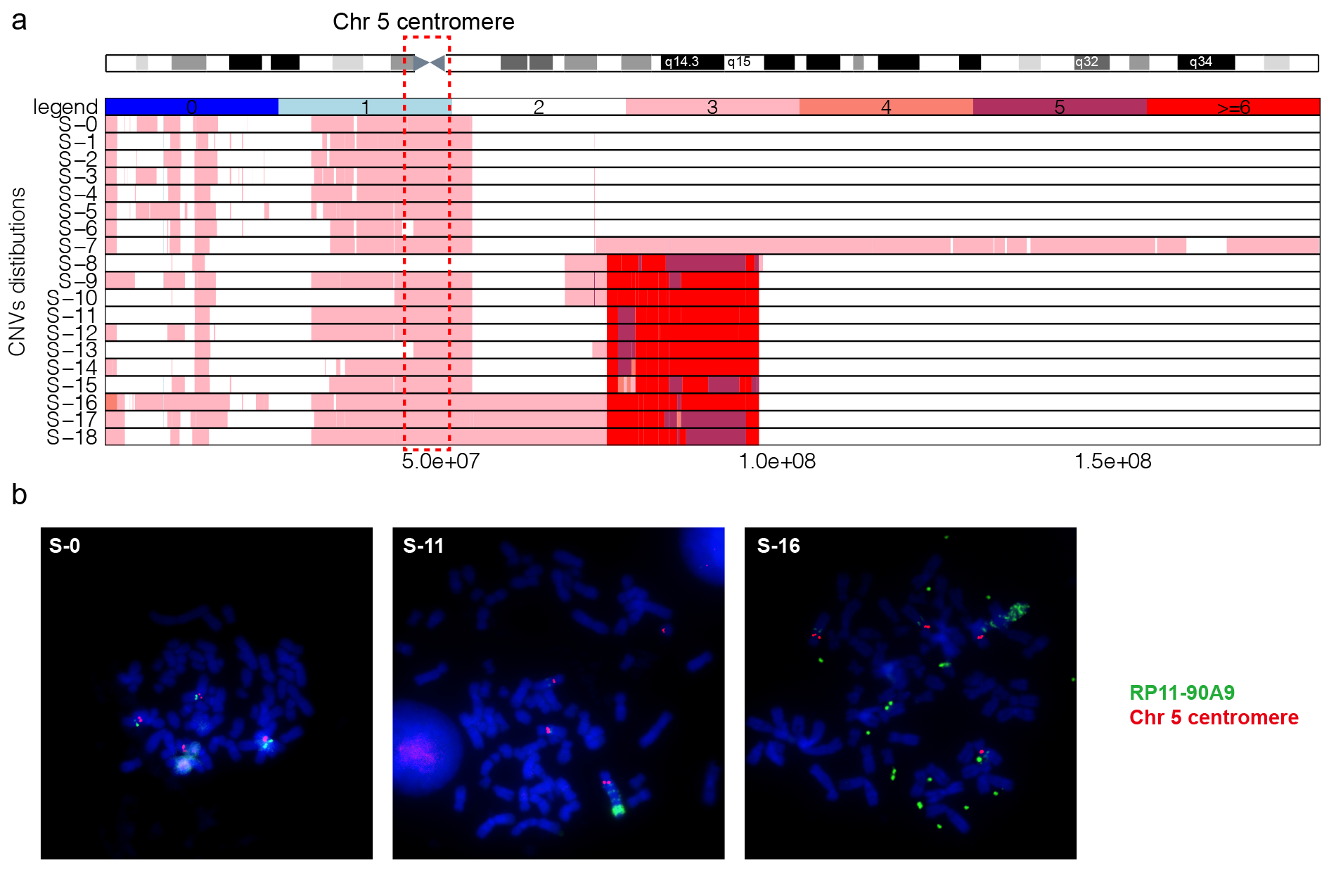
**Figure S7** Ploidy analysis of MTX resistant cells.

a. Copy number of the centromere region on chromosome 5 of MTX-resistant cells inferred from the NGS data. The NGS data showed that the copy number of chromosome 5 centromere region is near constant during all MTX resistant cells (S-0, S-1 to S-7, and S-9 to S-18) except S-8. b. Copy number of centromeric and *DHFR* gene on chromosome 5 of MTX-resistant cells. FISH results showed that the copy number of chromosome 5 centromere region did not change from pre-amplified (S-0), HSR (S-11) to ecDNA (S-16) stages, but the copy number of *DHFR* increased significantly.


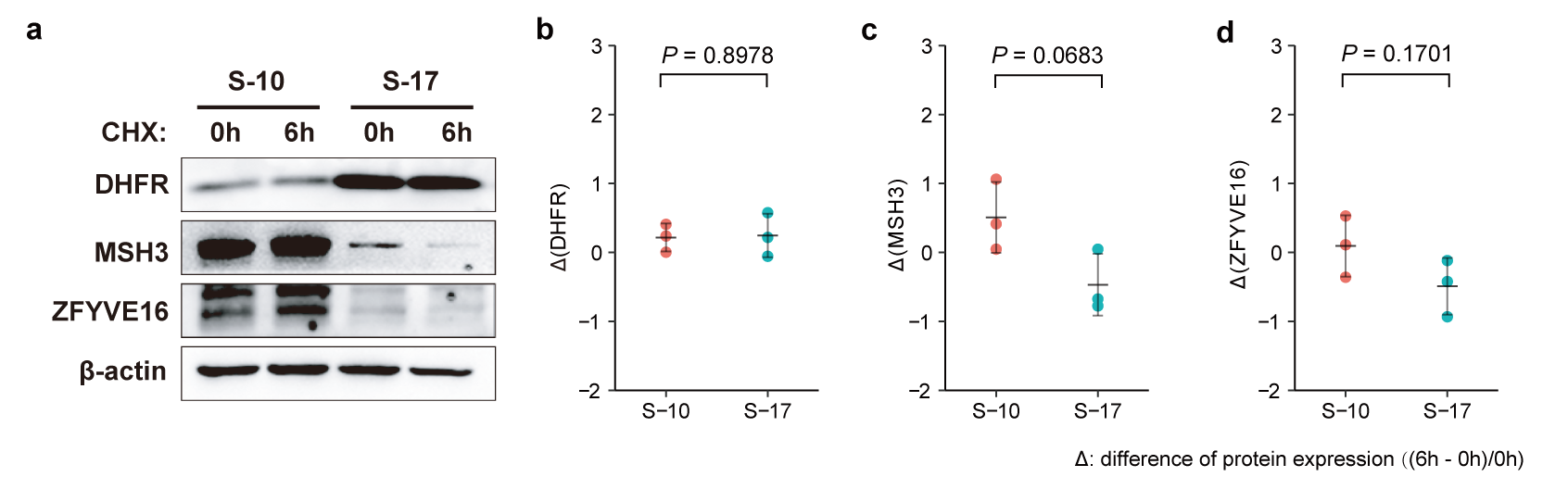


**Figure S8.** Effect of CHX on DHFR, MSH3 and ZFYVE16 expression in cells at HSR and ecDNA phases. Proteins expression were detected by WB after CHX (250μg/ml) treatment for 0 and 6 hours. To avoid the different expression background in S-10 (HSR) and S-17 (ecDNA), difference of protein level was normalized to the protein level at 0 h in each experiment. Data are presented by mean ± standard deviation and analyzed by One-way ANOVA.


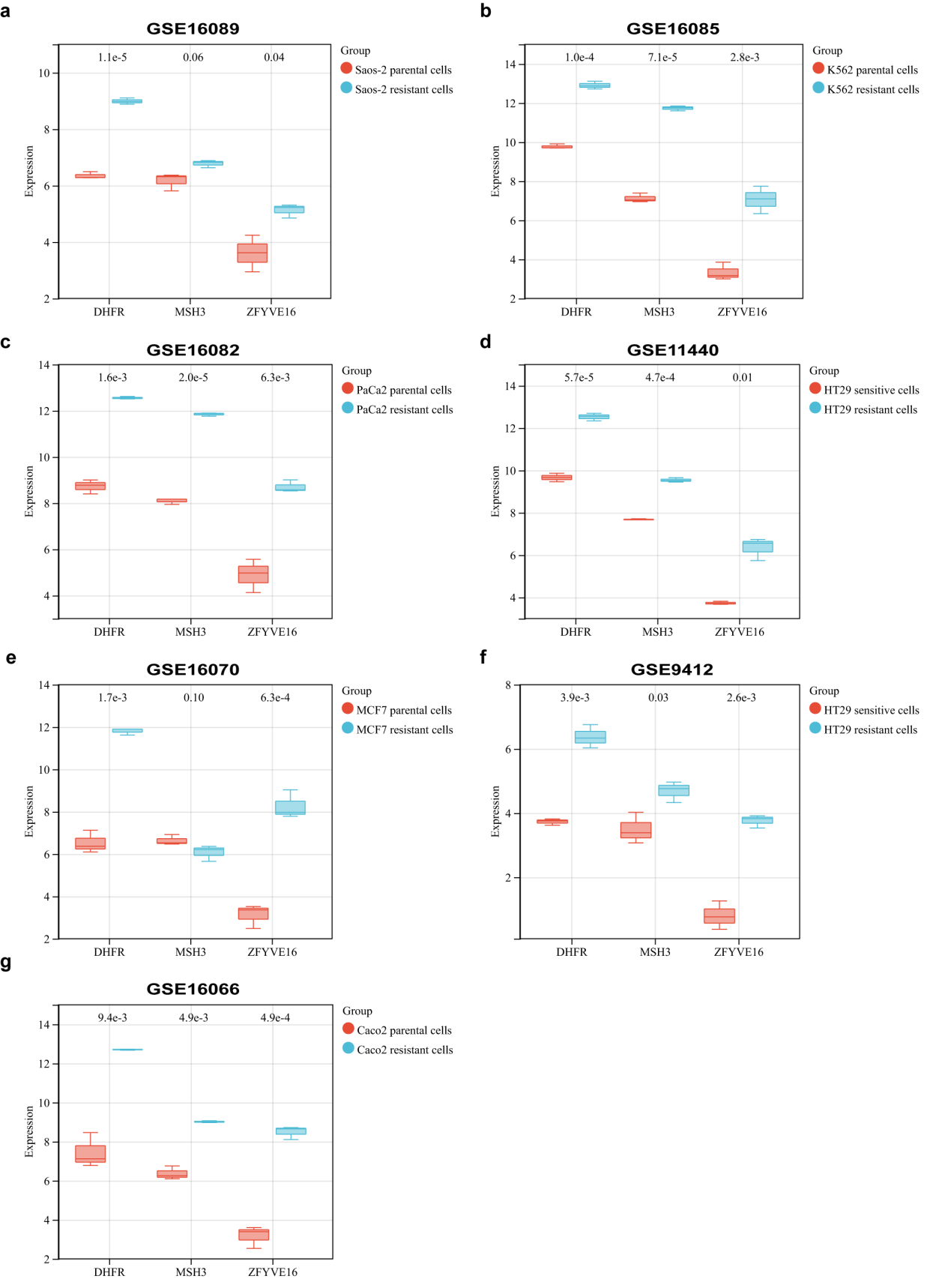


**Figure S9.** Expression of *DHFR*, *MSH3* and *ZFYVE16* in the different MTX-resistant cancer cell lines (Saos-2, K562, PaCa2, HT-29, MCF7 and Caco2).

GEO (https://www.ncbi.nlm.nih.gov/gds) transcriptome data were used to detect the expression of *DHFR*, *MSH3* and *ZFYVE16* in MTX-resistant cancer cell lines. Apart from *MSH3* in GSE16070, the expression of *DHFR*, *MSH3* and *ZFYVE16* were higher in 7 MTX-resistant cancer cell lines than in parent cells. The serial numbers of GEO are shown above the [histogram](javascript:;)s.


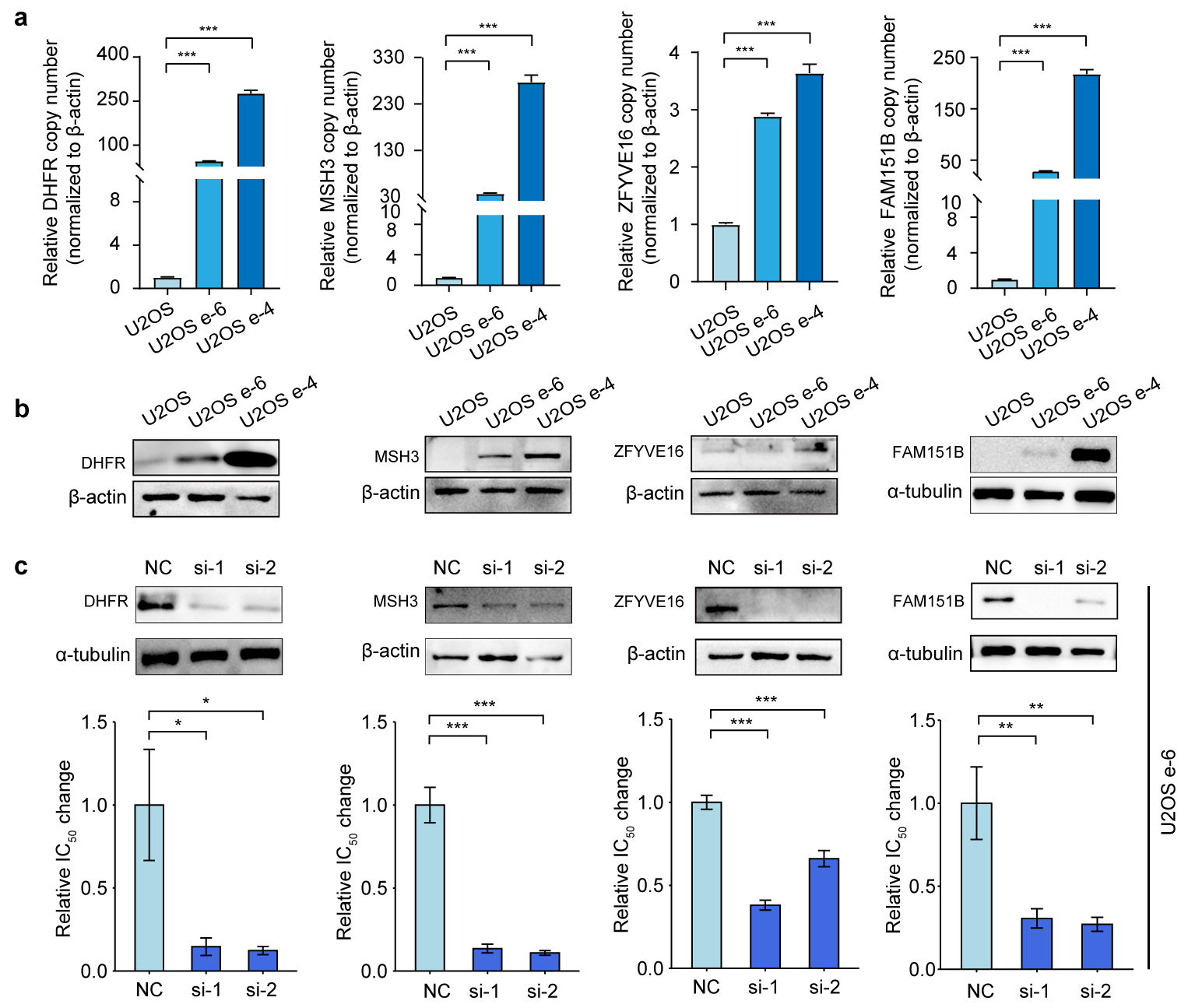


**Figure S10.** Functional analyses of *DHFR*, *MSH3*, *ZFYVE16* and *FAM151B* in the MTX-resistant U2OS cell lines.

a. Amplification of *DHFR*, *MSH3*, *ZFYVE16* and *FAM151B* in U2OS parental and MTX-resistant cells (U2OS e-6 and U2OS e-4, with MTX concentrations of 1.0×10^-6^ mol/L and 1.0×10^-4^ mol/L, respectively) (Student’s *t*-test, *** *P* < 0.001). b. Expression of *DHFR*, *MSH3*, *ZFYVE16* and *FAM151B* in U2OS parental and MTX-resistant cells (U2OS e-6 and U2OS e-4). c. Gene expression and relative IC_50_ change of control and two target-depleted cells (*DHFR*, *MSH3*, *ZFYVE16* and *FAM151b*) (Student’s *t*-test, ****P* < 0.001, ***P* < 0.01, **P* < 0.05).

Supplementary Tables

Table S1. IC_50_ values of HT29 and HT29 MTX-resistant cell lines

| Sample | MTX concentration | IC_50_ (mol/L)±SD | Fold change^A^ |
| --- | --- | --- | --- |
| S-0 | 0.0 | 7.49E-06±3.39E-07 | 1 |
| S-1 | 1.0e-7 | 1.25E-05±5.13E-06 | 1.67 |
| S-2 | 2.0e-7 | 5.14E-05±3.83E-06 | 6.86 |
| S-3 | 4.0e-7 | 6.85E-05±4.64E-06 | 9.15 |
| S-4 | 6.0e-7 | 8.75E-05±1.50E-05 | 11.68 |
| S-5 | 8.0e-7 | 4.41E-05±1.29E-06 | 5.89 |
| S-6 | 1.0e-6 | 9.36E-05±3.04E-05 | 12.5 |
| S-7 | 2.0e-6 | 1.41E-04±4.24E-05 | 18.83 |
| S-8 | 4.0e-6 | 4.82E-04±1.15E-04 | 64.35 |
| S-9 | 6.0e-6 | 6.48E-04±5.55E-05 | 86.52 |
| S-10 | 8.0e-6 | 5.09E-04±1.60E-04 | 67.96 |
| S-11 | 1.0e-5 | 4.94E-04±3.05E-05 | 65.95 |
| S-12 | 2.0e-5 | 6.68E-04±8.95E-05 | 89.19 |
| S-13 | 4.0e-5 | 6.28E-04±7.00E-05 | 83.85 |
| S-14 | 6.0e-5 | 1.53E-03±1.61E-04 | 204.27 |
| S-15 | 8.0e-5 | 2.24E-03±2.89E-04 | 299.07 |
| S-16 | 1.0e-4 | 3.37E-03±6.43E-04 | 449.93 |
| S-17 | 3.0e-4 | 4.12E-02±6.15E-03 | 5500.67 |
| S-18 | 6.0e-4 | 3.47E-02±2.54E-02 | 4632.84 |

A: IC_50_ of HT29 MTX-resistant / IC_50_ of HT29 cell line

IC_50_ values of MTX-resistant cell lines are highly correlated with increased MTX concentration (Pearson correction coefficient = 0.896, P value = 2.10 e-07), which confirms that MTX-resistant cell lines were successfully established.

Table S2. Statistics of whole genome sequences from all the cell lines from stage S-0 to S-18

| Sample | Total reads | Percentage of duplicate reads | Percentage of mapped reads | Percentage of PE reads | Percentage of SE reads | Mean sequence depth | Coverage≥ 1× | Coverage ≥ 4× | Coverage ≥ 10× | Coverage ≥ 20× |
| --- | --- | --- | --- | --- | --- | --- | --- | --- | --- | --- |
| S-0 | 786986664 (100%) | 9.42% | 99.29% | 99.24% | 0.10% | 36.00 | 98.98% | 98.65% | 97.07% | 86.32% |
| S-1 | 806366864 (100%) | 11.28% | 99.20% | 99.17% | 0.06% | 36.31 | 98.92% | 98.37% | 95.53% | 81.50% |
| S-2 | 698992424 (100%) | 9.92% | 99.38% | 99.34% | 0.08% | 31.96 | 98.95% | 98.41% | 95.06% | 76.50% |
| S-3 | 769128288 (100%) | 11.60% | 99.57% | 99.52% | 0.11% | 34.40 | 98.96% | 98.50% | 95.96% | 80.98% |
| S-4 | 700434284 (100%) | 9.83% | 99.76% | 99.71% | 0.11% | 32.01 | 98.96% | 98.54% | 96.13% | 79.65% |
| S-5 | 845073962 (100%) | 27.66% | 99.64% | 99.58% | 0.12% | 30.93 | 98.94% | 98.25% | 94.10% | 72.92% |
| S-6 | 738408246 (100%) | 10.29% | 99.73% | 99.67% | 0.12% | 33.58 | 98.97% | 98.54% | 96.04% | 79.94% |
| S-7 | 726040038 (100%) | 9.57% | 99.83% | 99.77% | 0.12% | 33.34 | 98.97% | 98.52% | 95.70% | 78.53% |
| S-8 | 718754550 (100%) | 14.03% | 99.56% | 99.52% | 0.09% | 31.36 | 98.93% | 98.26% | 94.00% | 73.04% |
| S-9 | 708618580 (100%) | 13.28% | 99.54% | 99.48% | 0.11% | 31.04 | 98.89% | 98.18% | 94.01% | 73.28% |
| S-10 | 722463938 (100%) | 10.97% | 99.64% | 99.61% | 0.06% | 32.80 | 98.91% | 98.40% | 95.78% | 80.69% |
| S-11 | 702838734 (100%) | 16.60% | 99.33% | 99.28% | 0.10% | 29.56 | 98.97% | 98.44% | 95.09% | 73.69% |
| S-12 | 745095018 (100%) | 27.98% | 99.75% | 99.70% | 0.11% | 27.19 | 98.92% | 98.17% | 93.28% | 66.92% |
| S-13 | 817229350 (100%) | 12.58% | 99.79% | 99.73% | 0.11% | 36.28 | 98.91% | 98.32% | 95.29% | 80.07% |
| S-14 | 713842138 (100%) | 11.93% | 98.79% | 98.73% | 0.11% | 31.65 | 98.94% | 98.45% | 95.56% | 77.07% |
| S-15 | 808770146 (100%) | 11.51% | 99.70% | 99.66% | 0.09% | 36.42 | 98.93% | 98.43% | 95.75% | 81.43% |
| S-16 | 780289530 (100%) | 14.14% | 98.67% | 98.63% | 0.08% | 33.72 | 98.95% | 98.54% | 96.32% | 82.65% |
| S-17 | 817711072 (100%) | 28.02% | 99.19% | 99.14% | 0.08% | 29.85 | 98.95% | 98.46% | 95.42% | 74.92% |
| S-18 | 702369052 (100%) | 11.34% | 99.28% | 99.25% | 0.05% | 31.68 | 98.89% | 98.39% | 95.75% | 78.99% |

PE: paired end; SE: single end.

The sequences were generated using 150bp paired end reads from the HiSeq X Ten platform

Table S3 Site and genotype concordance between NGS and SNP chip datasets in HT29 and HT29 MTX-resistant cell lines

| Sample | Unique CNVs | Confirmed by SNP chips | Confirmed by NGS | False negative rate of NGS | CNVs contain ≥5 probes in SNP chips | Detected by NGS and SNP chips | Site concordance | Detected by NGS and SNP chips with same CNV type | Genotype concordance |
| --- | --- | --- | --- | --- | --- | --- | --- | --- | --- |
| S-0 | 500 | 440 | 390 | 0.25 | 330 | 330 | 0.85 | 251 | 0.76 |
| S-1 | 537 | 493 | 419 | 0.24 | 379 | 375 | 0.90 | 291 | 0.78 |
| S-2 | 503 | 443 | 408 | 0.21 | 355 | 348 | 0.85 | 276 | 0.79 |
| S-3 | 535 | 476 | 434 | 0.21 | 380 | 375 | 0.86 | 282 | 0.75 |
| S-4 | 497 | 439 | 402 | 0.22 | 344 | 344 | 0.86 | 256 | 0.74 |
| S-5 | 539 | 457 | 429 | 0.24 | 372 | 347 | 0.81 | 267 | 0.77 |
| S-6 | 516 | 467 | 396 | 0.26 | 343 | 347 | 0.88 | 265 | 0.76 |
| S-7 | 526 | 463 | 426 | 0.22 | 372 | 363 | 0.85 | 280 | 0.77 |
| S-8 | 605 | 563 | 434 | 0.30 | 396 | 392 | 0.90 | 321 | 0.82 |
| S-9 | 521 | 466 | 424 | 0.21 | 377 | 369 | 0.87 | 275 | 0.75 |
| S-10 | 496 | 442 | 401 | 0.22 | 354 | 347 | 0.87 | 258 | 0.74 |
| S-11 | 502 | 445 | 400 | 0.23 | 347 | 343 | 0.86 | 266 | 0.78 |
| S-12 | 472 | 409 | 377 | 0.23 | 324 | 314 | 0.83 | 238 | 0.76 |
| S-13 | 499 | 442 | 418 | 0.18 | 373 | 361 | 0.86 | 273 | 0.76 |
| S-14 | 496 | 438 | 381 | 0.26 | 320 | 323 | 0.85 | 242 | 0.75 |
| S-15 | 472 | 414 | 376 | 0.23 | 332 | 318 | 0.85 | 237 | 0.75 |
| S-16 | 468 | 412 | 385 | 0.20 | 339 | 329 | 0.86 | 252 | 0.77 |
| S-17 | 528 | 476 | 423 | 0.22 | 381 | 371 | 0.88 | 291 | 0.78 |
| S-18 | 545 | 496 | 408 | 0.28 | 358 | 359 | 0.88 | 283 | 0.79 |
| Total | 9757 | 8681 | 7731 | 0.23 | 6776 | 6655 | 0.86 | 5104 | 0.77 |

Table S4. mtDNA copy number in cell lines of S-0 to S-18.

| Sample | Total_reads | MT_reads | Nuclear_reads | CN | Relative changes |
| --- | --- | --- | --- | --- | --- |
| S-0 | 789898197 | 1868039 | 788030158 | 0.00237 | 1.0000 |
| S-1 | 808463001 | 2762665 | 805700336 | 0.00343 | 1.44648 |
| S-2 | 701256108 | 2062446 | 699193662 | 0.00295 | 1.24435 |
| S-3 | 772546367 | 2128487 | 770417880 | 0.00276 | 1.16547 |
| S-4 | 703423485 | 1966236 | 701457249 | 0.00280 | 1.18247 |
| S-5 | 848541037 | 2666306 | 845874731 | 0.00315 | 1.32972 |
| S-6 | 741651062 | 1972142 | 739678920 | 0.00267 | 1.12474 |
| S-7 | 729176274 | 2087582 | 727088692 | 0.00287 | 1.21119 |
| S-8 | 721148013 | 2007779 | 719140234 | 0.00279 | 1.17777 |
| S-9 | 711714050 | 2271614 | 709442436 | 0.00320 | 1.35075 |
| S-10 | 724242422 | 1878259 | 722364163 | 0.00260 | 1.09687 |
| S-11 | 705746820 | 1715730 | 704031090 | 0.00244 | 1.02805 |
| S-12 | 748109378 | 1953808 | 746155570 | 0.00262 | 1.10461 |
| S-13 | 820444294 | 2148958 | 818295336 | 0.00263 | 1.10783 |
| S-14 | 716700734 | 1625064 | 715075670 | 0.00227 | 0.95868 |
| S-15 | 811399910 | 2325437 | 809074473 | 0.00287 | 1.21248 |
| S-16 | 782709134 | 2218206 | 780490928 | 0.00284 | 1.19892 |
| S-17 | 819962930 | 2469932 | 817492998 | 0.00302 | 1.27455 |
| S-18 | 704062320 | 2288473 | 701773847 | 0.00326 | 1.37564 |

Copy number = Total MT reads/Total nuclear reads; the copy number was called individually for each cell line.

Table S5 Continuous-changed MRS-CNVs

| Location | Band | Type | Samples | Groups | MTX-related genes |
| --- | --- | --- | --- | --- | --- |
| Chr1:247232000-249213000 | q44 | Amplification | S-11~S-15 | HSR |  |
| Chr2:213953000-214053000 | q34 | Amplification | S-10~S-18 | HSR, ecDNA |  |
| Chr5:54663000-74705000 | q11.2~13 | Amplification | S-16~S-18 | ecDNA |  |
| Chr5:74705000-97349000 | q13.3~15 | Amplification | S-8~S-18 | HSR, ecDNA | DHFR, MSH3 |
| Chr6:202000-76649000 | p, q11.1-14.1 | Deletion | S-4, S-6, S-8~S-15 | HSR | TPMT, HLA-G, HLA-E, XPO5, SLC29A1 |
| Chr6:162576000-170914000 | q26~27 | Deletion | S-16~S-18 | ecDNA | SOD2 |
| Chr7:1-7427000 | p22.1~22.3 | Amplification | S-1~S-15 | Pre-amplification, HSR |  |
| Chr7:21333000-159123000 | p15.3~11.1,q | Amplification | S-1~S-15 | Pre-amplification, HSR | ABCB1, CYP3A4, IMPDH1, NOS3 |
| Chr9:204000-36098000 | p13.3~24 | Deletion | S-16~S-18 | ecDNA |  |
| Chr9:71032000-141018000 | q21~34 | Deletion | S-16~S-18 | ecDNA | SLC28A3, TLR4, CDK9, FPGS, ENG |
| Chr12:120649000-120847000 | q24.24, q24.31 | Amplification | S-9~S-18 | HSR, ecDNA |  |
| Chr14:75387000-75460000 | q24.3 | Amplification | S-16~S-18 | ecDNA |  |

Table S6. Previously reported MTX-resistant genes in MRS-CNVs

| Gene |  | Position | Karyotype band | | Copy number | Cell lines |
| --- | --- | --- | --- | --- | --- | --- |
| DHFR |  | 5:79922044-79950800 | | 5q14.1 | ＞5 | S7~S18 |
| MSH3 |  | 5:79950466-80172634 | | 5q14.1 | ＞5 | S7~S18 |
| TPMT |  | 6:18128544-18155374 | | 6p22.3 | ＜2 | S4, S6, S9~S15 |
| HLA-G |  | 6:29794744-29798902 | | 6p22.1 | ＜2 | S4, S6, S9~S15 |
| HLA-E |  | 6:30457244-30461982 | | 6p21.33 | ＜2 | S4, S6, S9~S15 |
| XPO5 |  | 6:43490067-43543812 | | 6p21.1 | ＜2 | S4, S6, S9~S15 |
| SLC29A1 |  | 6:44187241-44201888 | | 6p21.1 | ＜2 | S4, S6, S9~S15 |
| SOD2 |  | 6:160100148-160114353 | | 6q25.3 | ＜2 | S1~S18 |
| ABCB1 |  | 7:87133178-87342639 | | 7q21.12 | 2＜ ≤5 | S1~S6, S9~S10, S12~S15 |
| CYP3A4 |  | 7:99354582-99381811 | | 7q22.1 | 2＜ ≤5 | S1~S18 |
| IMPDH1 |  | 7:128032330-128050036 | | 7q32.1 | 2＜ ≤5 | S1~S6, S8~S15 |
| NOS3 |  | 7:150688143-150711687 | | 7q36.1 | 2＜ ≤5 | S1~S18 |
| SLC28A3 |  | 9:86890764-86983413 | | 9q21.33 | ＜2 | S16~S18 |
| TLR4 |  | 9:120466452-120479769 | | 9q33.1 | ＜2 | S16~S18 |
| CDK9 |  | 9:130548304-130553052 | | 9q34.11 | ＜2 | S16~S18 |
| FPGS |  | 9:130556875-130576556 | | 9q34.11 | ＜2 | S16~S18 |
| ENG |  | 9:130577290-130617052 | | 9q34.11 | ＜2 | S16~S18 |

17 MTX-resistant genes were detected in MRS-CNVs, and their information is listed in this table.

Table S7. Details of genes located on chr5:79474000-80170000

| Chromosome | Start (bp) | End (bp) | Gene name | Gene type |
| --- | --- | --- | --- | --- |
| 5 | 79535306 | 79535408 | snoU13 | snoRNA |
| 5 | 79584268 | 79585600 | KRT18P45 | pseudogene |
| 5 | 79595497 | 79598666 | CTC-512J14.7 | pseudogene |
| 5 | 79602054 | 79602187 | SNORA31 | snoRNA |
| 5 | 79611490 | 79611645 | CTC-512J14.1 | pseudogene |
| 5 | 79615444 | 79617661 | SPZ1 | protein_coding |
| 5 | 79627392 | 79628360 | RBMX2P5 | pseudogene |
| 5 | 79646840 | 79647775 | CTC-512J14.5 | pseudogene |
| 5 | 79654899 | 79655859 | HNRNPA1P12 | pseudogene |
| 5 | 79661545 | 79661651 | RNU6-211P | snRNA |
| 5 | 79703832 | 79775169 | ZFYVE16 | protein_coding |
| 5 | 79778112 | 79783882 | CTD-2015H6.3 | antisense |
| 5 | 79783788 | 79838382 | FAM151B | protein_coding |
| 5 | 79794353 | 79794790 | CTD-2015H6.2 | pseudogene |
| 5 | 79796151 | 79797219 | RPL7P24 | pseudogene |
| 5 | 79852574 | 79866307 | ANKRD34B | protein_coding |
| 5 | 79899202 | 79899463 | DBIP2 | pseudogene |
| 5 | 79904442 | 79918343 | CTC-325J23.3 | lincRNA |
| 5 | 79922047 | 79950802 | DHFR | protein_coding |
| 5 | 79926132 | 79927409 | CTC-325J23.2 | antisense |
| 5 | 79945819 | 79946855 | MTRNR2L2 | protein_coding |
| 5 | 79950467 | 80172279 | MSH3 | protein_coding |
| 5 | 80042158 | 80042638 | RP11-241J12.3 | pseudogene |
| 5 | 80151326 | 80151662 | RP11-241J12.1 | pseudogene |

24 genes were detected within chr5: 79474000-80170000. 7 of them are protein coding genes: *SPZ1, ZFYVE16, FAM151B, ANKRD34B, DHFR, MTRNR2L2* and *MSH3*.

Table S8. mAF ranges of segments with different copy numbers

| Ploid | NOMA | PmAF | SmAF | LmAF | ImAF |
| --- | --- | --- | --- | --- | --- |
| 2 | 1 | 0.50 | 0.43~0.50 | 0.44~0.50 | 0.44~0.50 |
| 3 | 1 | 0.33 | 0.27~0.36 | 0.31~0.38 | 0.31~0.36 |
| 4 | 1 | 0.25 | 0.20~0.27 | 0.24~0.29 | 0.24~0.27 |
| 4 | 2 | 0.50 | 0.47~0.50 | 0.47~0.50 | 0.47~0.50 |
| 5 | 1 | 0.20 | 0.16~0.21 | 0.19~0.24 | 0.19~0.21 |
| 5 | 2 | 0.40 | 0.37~0.42 | 0.38~0.43 | 0.38~0.42 |
| 6 | 1 | 0.17 | 0.13~0.17 | 0.16~0.20 | 0.16~0.17 |
| 6 | 2 | 0.33 | 0.30~0.35 | 0.32~0.36 | 0.32~0.35 |
| 6 | 3 | 0.50 | 0.48~0.50 | 0.48~0.50 | 0.48~0.50 |
| 7 | 1 | 0.14 | 0.11~0.15 | 0.14~0.17 | 0.14~0.15 |
| 7 | 2 | 0.29 | 0.26~0.30 | 0.28~0.31 | 0.28~0.30 |
| 7 | 3 | 0.43 | 0.41~0.44 | 0.41~0.45 | 0.41~0.44 |
| 8 | 1 | 0.12 | 0.10~0.13 | 0.12~0.15 | 0.12~0.13 |
| 8 | 2 | 0.25 | 0.23~0.26 | 0.24~0.27 | 0.24~0.26 |
| 8 | 3 | 0.38 | 0.35~0.39 | 0.36~0.39 | 0.36~0.39 |
| 8 | 4 | 0.50 | 0.48~0.50 | 0.48~0.50 | 0.48~0.50 |

NOMA: number of one minor allele of the major subclone;

PmAF: mAF of pure cell line (no subclones);

SmAF: mAF of major subclone mixed with a minor subclone with lower copy number;

LmAF: mAF of major subclone mixed with a minor subclone with higher copy number;

ImAF: integrated mAF of SmAF and LmAF. The mixture was considered as a major subclone and a minor subclone, and the integrated mAF ranges would close to PmAF value.

**Note:** Cancer cell lines usually consist of many different subclones, so we simplified the complex situation as a mixture of a major and minor subclones and improved the formula proposed by Popova *et al.* to get the final mAF ranges as below^4^:

where n_B_ was the number of B allele, the minor allele; n_A_ was number of A allele, c1 and c2 were two subclones, p was the occupation of c2 subclone and was set to 0.6 based on the accuracy of the SNP chips^5^.

Because of the saturation effect of chips and methods we used, we only focused on the theoretical mAF ranges corresponding to the copy numbers of 0 to 8^6^, as it is difficult to distinguish signal from noise when the copy number was more than 8. In addition, HT29 and MTX-resistant HT29 cell lines were nearly triploid, so mAF of points with mLRR around 0 were about 0.33.

Table S9. mLRR ranges of segments with different copy numbers

| Sample | CN=2 | | CN=3 | | CN=4 | | CN=5 | |
| --- | --- | --- | --- | --- | --- | --- | --- | --- |
|  | Start | End | Start | End | Start | End | Start | End |
| S-0 | -0.39 | -0.09 | -0.06 | 0.05 | 0.07 | 0.24 | 0.26 | 0.29 |
| S-1 | -0.39 | -0.19 | -0.09 | 0.07 | 0.08 | 0.21 | 0.23 | 0.30 |
| S-2 | -0.31 | -0.18 | -0.08 | 0.08 | 0.09 | 0.20 | 0.21 | 0.28 |
| S-3 | -0.41 | -0.19 | -0.09 | 0.06 | 0.10 | 0.22 | 0.27 | 0.32 |
| S-4 | -0.40 | -0.19 | -0.06 | 0.06 | 0.10 | 0.22 | 0.27 | 0.30 |
| S-5 | -0.44 | -0.2 | -0.11 | 0.07 | 0.04 | 0.23 | 0.25 | 0.32 |
| S-6 | -0.35 | -0.19 | -0.06 | 0.06 | 0.10 | 0.21 | 0.24 | 0.28 |
| S-7 | -0.44 | -0.19 | -0.10 | 0.07 | 0.06 | 0.21 | 0.27 | 0.31 |
| S-8 | -0.40 | -0.22 | -0.10 | 0.06 | 0.10 | 0.20 | 0.24 | 0.31 |
| S-9 | -0.38 | -0.22 | -0.08 | 0.06 | 0.10 | 0.21 | 0.26 | 0.32 |
| S-10 | -0.38 | -0.23 | -0.06 | 0.06 | 0.10 | 0.21 | 0.26 | 0.30 |
| S-11 | -0.41 | -0.21 | -0.08 | 0.05 | 0.08 | 0.22 | 0.23 | 0.29 |
| S-12 | -0.38 | -0.18 | -0.11 | 0.05 | 0.11 | 0.22 | 0.21 | 0.26 |
| S-13 | -0.47 | -0.18 | -0.08 | 0.10 | 0.09 | 0.20 | 0.24 | 0.30 |
| S-14 | -0.41 | -0.21 | -0.14 | 0.09 | 0.06 | 0.23 | 0.26 | 0.33 |
| S-15 | -0.45 | -0.20 | -0.11 | 0.06 | 0.10 | 0.22 | 0.26 | 0.32 |
| S-16 | -0.37 | -0.11 | -0.06 | 0.07 | 0.10 | 0.20 | 0.26 | 0.31 |
| S-17 | -0.45 | -0.16 | -0.10 | 0.08 | 0.10 | 0.25 | 0.29 | 0.34 |
| S-18 | -0.48 | -0.15 | -0.14 | 0.10 | 0.08 | 0.24 | 0.26 | 0.31 |

The mLRR ranges of segments with different copy numbers were confirmed by manually viewing the cluster results shown in Supplementary Figure S1, and the final copy numbers were also confirmed by a manual check.

Table S10. Primer sequences used in qPCR for the genes on chr5:79474000-80170000

| Gene | primer | DNA Sequence (5´-3´) | cDNA Sequence (5´-3´) |
| --- | --- | --- | --- |
| DHFR | F | ATTTTGTTCAGTGCCTACCACA | CCGGCACATCTTCATTCTTT |
|  | R | GCCTGAATGATATCTACAAGCTG | GCAACCATCATCCCTCACTT |
| MSH3 | F | TGTCTGGTG TTTCGCCTGAT | CTGCCAAAGTTGGGGATAAA |
|  | R | TTAGCCAATAACCGCTCTA | AAATGCATTCGGATCTCGTC |
| ZFYVE16 | F | AG GAAGCAACCACCACAAC | CCGAGCATGTGGGAAAGTATT |
|  | R | CAGCACCACCAACAGATACA | TCCTTTCAAATGCCTGAGCTT |
| FAM151B | F | ATTTCAGCCGACCGTATGGG | TTCCCTGGGTTGGACAACAG |
|  | R | AAGCACCTGATTCCAAGGGG | AGTCAGGCTGTACCTGTTTGAT |
| ANKRD34B | F | TGGATGGTGATCGCCTTAGC | GAGGACTAGTAGGAACTGACAGC |
|  | R | GGCGCGTTTCCTCAGTTTTT | GGATCAGTTGACAGCTTGGGT |
| SPZ1 | F | AAGGCTGTCTGAGATGCCAC | AAGTCAGCTGAGATGCCCAC |
|  | R | TTAGCAGAGCTGGCCATCAG | TCTGTGCAGTCTGTTGTTCTGT |
| MTRNR2L2 | F | GAACCCTCCAAATCCCCCTG | AACCCTCCAAATCCCCCTGTA |
|  | R | GGGTGCAAGGAGTTCAGTCA | CAGTCATATGTTTGGGATTTTTCGG |
| β-actin | F | TTCTGCCGTTTTCCGTAGG | AAATCTGGCACCACACCTTC |
|  | R | TTGGGATGGGGAGTCTGTT | GGGGTGTTGAAGGTCTCAAA |

F: forward; R: reverse

Table S11. Antibodies for proteins on chr5:79474000-80170000

| Gene | Antibody | Vendor | Catalog number |
| --- | --- | --- | --- |
| SPZ1 | rabbit polyclonal antibody | Abcam, Cambridge, UK | ab235302 |
| ZFYVE16 | rabbit polyclonal antibody | Thermo Fisher Scientific, MA, USA | PA5-25044 |
| FAM151B | rabbit polyclonal antibody | Thermo Fisher Scientific, MA, USA | PA5-60518 |
| ANKRD34B | rabbit polyclonal antibody | Thermo Fisher Scientific, MA, USA | PA5-60326 |
| DHFR | mouse monoclonal antibody | Abnova, Taipei, Taiwan | MAB22083 |
| MSH3 | mouse monoclonal antibody | BD Transduction Laboratories, San Diego, CA, USA | 611390 |
| GAPDH | mouse monoclonal antibody | Kang Chen Bio-tech, Shanghai, China | KC-5G4 |
| Beta Actin | mouse monoclonal antibody | Proteintech, Wuhan, China | 66009-1-Ig |
| Alpha Tubulin | rabbit polyclonal antibody | Proteintech, Wuhan, China | 11224-I-AP |
| IRDye 800 | anti- rabbit IgG | Rockland, Limerick, PA, USA | 611-132-122 |
| IRDye 700 | anti-mouse IgG | Rockland, Limerick, PA, USA | 610-730-124 |

Table S12. siRNA sequences of *DHFR*, *MSH3*, *ZFYVE16* and *FAM151B*.

| siRNA | Sequences (5´-3´) |
| --- | --- |
| NC-si  DHFR-si-1  DHFR-si-2  MSH3-si-1  MSH3-si-2 | UUCUCCGAACGUGUCACGUTT  GAGAAGAAUCGACCUUUAAAGTT  GUUGGUUCGCUAAACUGCAUCTT  CUUCUACCAGCUAUCUUCUTT  AGAGCCACAUCUGUUAGUGTT |
| ZFYVE16-si-1  ZFYVE16-si-2  FAM151B-si-1  FAM151B-si-2 | GGAAGAUAAGACUGUUAUAAATT  GGACGAUGUAGUAAACCUAUCT  GGAUUAAUGCCGAUAUUCUUCTT  GGAAUUCAAAGUUAAUCUCUAATT |

References

1. Pique-Regi R, Cáceres A, González JR. R-Gada: a fast and flexible pipeline for copy number analysis in association studies. BMC Bioinformatics. 2010;11:380

2. Pique-Regi R, Monso-Varona J, Ortega A, Seeger RC, Triche TJ, Asgharzadeh S. Sparse representation and Bayesian detection of genome copy number alterations from microarray data. Bioinformatics. 2008;24(3):309-18.

3. Attiyeh EF, Diskin SJ, Attiyeh MA, Mossé YP, Hou C, Jackson EM, et al. Genomic copy number determination in cancer cells from single nucleotide polymorphism microarrays based on quantitative genotyping corrected for aneuploidy. Genome Res. 2009;19(2):276-83.

4. Popova T, Manié E, Stoppa-Lyonnet D, Rigaill G, Barillot E, Stern MH. Genome Alteration Print (GAP): a tool to visualize and mine complex cancer genomic profiles obtained by SNP arrays. Genome Biol. 2009;10(11):R128.

5. Peiffer DA, Le JM, Steemers FJ, Chang W, Jenniges T, Garcia F, et al. High-resolution genomic profiling of chromosomal aberrations using Infinium whole-genome genotyping. Genome Res. 2006;16(9):1136-48.

6. Conrad DF, Andrews TD, Carter NP, Hurles ME, Pritchard JK. A high-resolution survey of deletion polymorphism in the human genome. Nat Genet. 2006;38(1):75-81.
